# UPLC-PDA factorial design assisted method for simultaneous determination of oseltamivir, dexamethasone, and remdesivir in human plasma

**DOI:** 10.1038/s41598-024-71413-3

**Published:** 2024-09-18

**Authors:** Hanan I. EL-Shorbagy, Mona A. Mohamed, Alaa El-Gindy, Ghada M. Hadad, Fathalla Belal

**Affiliations:** 1 Pharmaceutical Analytical Chemistry Department, Faculty of Pharmacy, Suez Canal University, Ismailia, 41522 Egypt; 2 Pharmaceutical Chemistry Department, Egyptian Drug Authority (EDA), Cairo, Egypt; 3 Department of Pharmaceutical Analytical Chemistry, Faculty of Pharmacy, Mansoura University, Mansoura, 35516 Egypt

**Keywords:** UPLC-PDA, Fluoride-EDTA plasma, Gibbs free energy, Green analysis, Environmental sciences, Bioanalytical chemistry

## Abstract

A green and simple UPLC method was developed and optimized, adopting a factorial design for simultaneous determination of oseltamivir phosphate and remdesivir with dexamethasone as a co-administered drug in human plasma and using daclatasvir dihydrochloride as an internal standard within 5 min. The separation was established on UPLC column BEH C_18_ 1.7 μm (2.1 $\times$ 100.0 mm) connected to UPLC pre-column BEH 1.7 μm (2.1 $\times$ 5.0 mm) at 50 °C with an injection volume of 10 μL. The photodiode array detector (PDA) was set at three wavelengths of 220, 315, and 245 nm for oseltamivir phosphate, the internal standard, and both dexamethasone and remdesivir, respectively. The mobile phase consisted of methanol and ammonium acetate solution (40 mM) adjusted to pH 4 in a ratio of 61.5:38.5 (v/v) with a flow rate of 0.25 mL min^−1^. The calibration curves were linear over 500.0–5000.0 ng mL^−1^ for oseltamivir phosphate, over 10.0–500.0 ng mL^−1^ and 500.0–5000.0 ng mL^−1^ for dexamethasone, and over 20.0–500 ng mL^−1^ and 500.0–5000.0 ng mL^−1^ for remdesivir. The Gibbs free energy and Van't Hoff plots were used to investigate the effect of column oven temperatures on retention times. Fluoride-EDTA anticoagulant showed inhibition activity on the esterase enzyme in plasma. The proposed method was validated according to the M10 ICH, FDA, and EMA’s bioanalytical guidelines. According to Eco-score, GAPI, and AGREE criteria, the proposed method was considered acceptable green.

## Introduction

COVID-19 created a serious threat to economies and healthcare systems. SARS-CoV-2 viruses, which are encapsulated viruses with a single-stranded RNA genome, are the main cause of COVID-19^1,2^. In the management protocol for COVID-19, oseltamivir phosphate (OSTP) (Fig. S1A) is indicated for mild cases, while remdesivir (REM) (Fig. S1C) is indicated for moderate-to-severe cases^3–10^. It was indicated that dexamethasone (DEX) (Fig. S1B), as an anti-infamatory glucocorticoid, is coadministered with OSTP or REM in COVID-19 viral infections. The U.S. Food and Drug Administration (FDA) authorized OSTP in August 2016^11^. It stops viral budding from the host cell by inhibiting the neuraminidase enzyme on the surface of the virus. It is used to both treat and prevent influenza virus infections. The FDA authorized REM for the treatment of COVID-19 in October 2020^7–9^. It is an analog of a nucleoside that inhibits the viral polymerase enzyme, preventing the replication of the virus.

According to literatures, OSTP was estimated by capillary zone electrophoresis (CE), chromatographic, mass spectrometric (MS), enzymatic, colorimetric, spectrophotometric, and spectrofluorimetric methods since its FDA authorization until 2011^12,13^. Since 2011, OSTP has been determined by RP-HPLC^14–16^, UPLC^17^, LC–MS /MS^18,19^, spectrofluorimetric^20^, mass spectrometry (MS)^21^, potentiometric^22^, and spectrophotometric^23^ methods. DEX was estimated by spectrophotometric, UPLC-UV, HPLC–UV, and LC–MS/MS techniques since its FDA authorization until 2022^24^. REM was determined by UPLC^25^, LC–MS/MS^26^, RP-HPLC^27^, electrochemical^28^, and spectrofluorimetric^29^ methods. No analytical method has yet been reported to determine OSTP, DEX, and REM simultaneously in human plasma. So, it was important to develop a method that is fast, accurate, and green to simultaneously separate OSTP, DEX, and REM from human plasma samples to ease the management of these drugs during the treatment of the huge numbers of COVID-19 patients.

The UPLC method was the first candidate method due to its several advantages, including its ability to increase chromatographic efficiency and sensitivity, improve analyte resolution, decrease run time, and lower solvent consumption^30^.

Several significant concerns were carefully investigated during this work. Before application to human plasma, this suggested method was initially optimized by means of the design of experiments (DOE) technique at a column oven temperature of 25 °C. Full factorial designs with one center-point experiment were used to determine the curvature possibilities (lack of fit) of the selected dependent responses, followed by multiple regression analysis. The Gibbs free energy (G°) of the solute interactions with the stationary phase was then used to investigate the effect of column oven temperatures of 25, 30, 45, and 50 °C on the retention times of the studied drugs in reversed-phase chromatography. The proposed method was validated according to the M10 ICH^31^, FDA^32^, and EMA’s^33^ bioanalytical method validation guidelines^31^. The bench-top (short-term) stability of each drug at room temperature for 2 h in different types of human plasma samples withdrawn into fluoride, K_3_EDTA, and fluoride-EDTA tubes was investigated. In addition, their matrix effect, extraction efficiency, process efficiency, and internal standard normalized values were estimated according to the M10 ICH's bioanalytical method validation guidelines^31^.

The proposed method has several advantages over other reported LC methods^14,17,25,27^. It is appropriate for UPLC-MS method development since the mobile phases utilized (methanol and ammonium acetate) are compatible with the ionization within mass detectors. In UPLC-MS, methanol is a safe solvent, while ammonium acetate is a volatile salt that is often used to buffer mobile phases. According to the lack of fit test of the factorial design experiments, the effects of methanol percentage, flow rate, or ammonium acetate concentration against k' (OSTP) or R_S2_ (DAC) are linear (no curvature possibilities), indicating good precision and easy robustness prediction of the proposed method. Fluoride-EDTA human plasma showed inhibition activity on the esterase enzyme in plasma, hence the highest stability results for the studied drugs. The proposed method is simple in sample preparation and extraction. Furthermore, the suggested method's greenness was evaluated using AGREE (Analytical GREEnness) analysis^34^, Eco-Scale^35^, and the Green Analytical Procedure Index (GAPI) criteria^36^.

## Experimental

### Apparatus and software

The developed method was carried out on an ACQUITY UPLC H-Class PLUS System (USA) equipped with a Waters quaternary solvent manager (M20QSP 471A), a Waters column heater (K20CHA 249G) with an UPLC column BEH C_18_ (1.7-μm, 2.1 $\times$ 100 mm) connected to an UPLC pre-column BEH (1.7-μm Van Guard, 2.1 $\times$ 5 mm), a Waters temperature-controlled sample manager-flow-through needle (SM-FTN-H), and a PDA detector (L20UPL 182A). The data was recorded and analyzed using the WatersEmpower3 chromatography software. For the preparation of the mobile phase, a water deionizer (Stakpure-OmniaLab, Germany), a balance (Sartorius, Germany), a pH meter (Jenway 3510, UK), an ultrasonicator (Elma, Germany), a vortex mixer (WiseMix, Portugal), a vacuum pump (Rocker 400, Taiwan), and nylon membrane filters (47 mm in diameter, 0.22 μm pore size, Chromtech, UK) were used. Pro manual Pipet4u micropipettes (Biotechnologie GmbH, Germany) were used for aliquot transfer during the preparation of the samples. An ultra-low-temperature freezer (Binder GmbH, Germany), a tabletop centrifuge (Harmonic series, Taiwan), and a thermostatic water bath (Gemmy Industrial Crop, Taiwan) were used for the preservation and preparation of plasma samples. All extracted samples were filtered using syringe filters (13 mm in diameter, 0.22 μm pore size, Chromtech PTFE, UK) before automatic injection into the UPLC chromatograph. Minitab Statistical Software (Release 16^37^, State College, Pennsylvania, USA) was used for the factorial design statistical analyses.

### Materials and solvents

The standard powders of OSTP (purity: 99.976% w/w), DEX (purity: 99.307% w/w), REM (purity: 99.841% w/w), and the internal standard daclatasvir dihydrochloride (DAC) (purity: 101.171% w/w) were provided by NODCAR, Cairo, Egypt. Methanol (HPLC grade, Fisher Scientific, Germany, purity: ≥ 99.9%), ammonium acetate (AA) (Oxford laboratory reagent, India, purity: 96%), and diethyl ether (laboratory reagent grade, UK) were bought from Cornell Lab-Fine Chemicals & Lab Equipments, Cairo, Egypt. K_3_EDTA (Golden Vac, Turkey) and glucose/fluoride (Golden Vac, Turkey) blood tube kits were purchased from local laboratory stores. Human blood samples were kindly donated individually by four adult females: volunteer A (aged 21 years old, donated 10 mL of blood), volunteer B (aged 24 years old, donated 5 mL of blood), volunteer C (aged 30 years old, donated 5 mL of blood), and volunteer D (aged 37 years old, donated 135 mL of blood). From the collected blood, nearly 80 mL of plasma was obtained, from which nearly 155 samples were prepared. We confirm that all experiments were performed in accordance with relevant guidelines and regulations. The study was conducted according to the rules of the Ethics Committee at the Faculty of Pharmacy, Suez Canal University (number: 202009PHDH1). Informed consent was obtained from all subjects and/or their legal guardian(s).

### Standard solutions

Four stock solutions of OSTP, DEX, REM, and DAC were each prepared in a volumetric flask (10 mL) and completed to the mark with methanol (HPLC grade, ≥ 99.9%) as a solvent. Each stock solution contained 500 μg mL^−1^ for each drug. Two different working ternary solutions of OSTP, DEX, and REM were prepared from their methanolic stock solutions. Each working ternary solution was prepared in 10-mL volumetric flasks and diluted with methanol (HPLC grade, ≥ 99.9%). The first methanolic working ternary solution contained 50.0 μg mL^−1^ of each drug, while the second methanolic working ternary solution contained 5.0 μg mL^−1^ of each drug. From the methanolic DAC stock solution, an internal standard (IS) (50.0 μg mL^−1^ DAC) was prepared in a volumetric flask (10 mL) and completed to the mark with methanol (HPLC grade, ≥ 99.9%) as a solvent. All prepared methanolic solutions were preserved in the freezer (from – 15 °C to − 28 °C) and protected from light.

### General recommended procedures

We confirm that all experiments were performed in accordance with relevant guidelines and regulations. The study was conducted according to the rules of the Ethics Committee at the Faculty of Pharmacy, Suez Canal University (number: 202009PHDH1). Informed consent was obtained from all subjects and/or their legal guardian(s).

#### Preparation of human fluoride-EDTA plasma

Human blood samples were withdrawn into K_3_-EDTA tubes (2 mL per tube) and immediately transferred into sodium fluoride tubes to prepare human fluoride-EDTA plasma. The tubes were then left for 5 min, then centrifuged at 3000 rpm for 10 min. The layers of separated plasma were then transferred into Eppendorf tubes and preserved in an ultralow freezer (− 80 °C).

#### Construction of calibration curves in human plasma

Ternary standard concentrations of OSTP, DEX, and REM ranging from 10.0 ng mL^−1^ to 5000.0 ng mL^−1^ for each drug were first prepared to estimate the linearity ranges of each drug. Different aliquots (ranging from 1 to 50 μL by micropipette) of the methanolic working ternary solution (5 μg mL^−1^ of each drug) were transferred into a series of stoppered test tubes to prepare ternary standard mixtures ranging from 10.0 ng mL^−1^ to 500.0 ng mL^−1^ for each drug in human plasma. Also, ternary standard concentrations ranging from 500.0 ng mL^−1^ to 5000.0 ng mL^−1^ for each drug were prepared by transferring different aliquots (ranging from 5 to 50 μL by micropipette) of the working ternary solution (50.0 μg mL^−1^ of each drug) into a series of stoppered test tubes. After the evaporation of methanol solvent at room temperature, 500 μL of plasma samples were added to the residues in each stoppered test tube and vortexed for 30 s. To each tube, 5 μL aliquots of the internal standard (50.0 μg mL^−1^) were added to the spiked plasma, then vortexed for 30 s. Finally, 2 mL aliquots of diethyl ether (DEE) were added to each tube, tightly closed, and vortexed for 1 min. The tubes were centrifuged at 3500 *rpm* for 5 min, and then 1.5 mL aliquots of the supernatant from each stoppered test tube were separately transferred into tailed test tubes and evaporated in a water bath at 40 °C. Finally, the produced residue was reconstituted in 100 μL of 60% (v/v) methanol, vortexed for 30 s, and filtrated using a membrane filter (pore size of 0.22 μm). Volume (10 μL) was analyzed (n = 6) under the suggested chromatographic conditions. The regression equations were generated by plotting the drug peak area to IS peak area ratios against their respective final concentrations.

#### Assessment of bench-top (short-term) stability

Aliquots of the methanolic working ternary solutions (50.0 μg mL^−1^ and 5.0 μg mL^−1^ of each drug) were transferred into a series of stoppered test tubes to prepare ternary standard solutions containing 1000.0 ng mL^−1^ and 300.0 ng mL^−1^ of each drug in plasma, respectively. After evaporation of methanol solvent at room temperature, 500 μL of fresh human plasma samples were added to the residue in each stoppered test tube and vortexed for 30 s. Each concentration, consisting of nine stoppered test tubes, was divided into three groups depending on the time of analysis: 0, 1, and 2 h. At the recommended time interval for each group, 5 μL aliquots of the IS (50.0 μg mL^−1^) were added to each test tube, and then the test tubes were vortexed for 30 s. Then they were extracted by the same procedure under Section “construction of calibration curves in human plasma”. Volumes of 10 μL were injected under optimal chromatographic conditions after filtration using 0.22 μm membrane filters. The peak areas were obtained, and the stability was estimated.

#### Estimation of matrix effect, extraction and process efficiency and their IS normalized values

For this test, three groups of samples (n = 3 for each group) were analyzed using four blank plasma samples (plasma A, B, C, and D) collected from different sources. The first group, known as Pre-Extraction Samples (PrES), contained extracted spiked human plasma; the second group, known as Post-Extraction Samples (PoES), contained extracted blank human plasma that was then spiked with the tested drugs and IS at the predicted final concentrations; and the third group, known as Solvent Samples (SS), contained the tested drugs and IS diluted in 60% (v/v) methanol at the predicted final concentrations. Two quality control levels were tested (1000.0 ng mL^−1^ and 300.0 ng mL^−1^ for each drug) in human plasma samples.

In the first group (PrES), aliquots of the working ternary solution (50.0 μg mL^−1^ of each drug) were transferred into a series of stoppered test tubes to prepare ternary standard concentrations of OSTP, DEX, and REM of 1000.0 ng mL^-1^ for each drug in human plasma. After evaporation of methanol solvent at room temperature, 500 μL of human plasma was added to the residues in each stoppered test tube, which was then vortexed for 30 s. Then, 5 μL of the IS (50.0 μg mL^−1^) was added to each one, and all stoppered test tubes were vortexed for 30 s. Then they were extracted by the same procedure under Section “Construction of Calibration Curves in Human Plasma." Finally, the produced residue was reconstituted in 100 μL of 60% (v/v) methanol for each stoppered test tube and vortexed for 30 s. In the second group (PoES), 500 μL of human plasma was added to each stoppered test tube and then extracted by the same procedure described under Section “Construction of Calibration Curves in Human Plasma." Finally, aliquots of 10 μL from the working ternary solution (50.0 μg mL^−1^ of each drug) and 5 μL from the IS (50.0 μg mL^−1^) were transferred into each of the evaporated-tailed test tubes and then completed to 100 μL by 60% (v/v) methanol. The final reconstitutions were then vortexed for 30 s. The third group (SS) was for the tested drugs diluted in 60% (v/v) methanol. The concentrations of the drugs in this quaternary standard mixture were 5.0 μg mL^−1^ for OSTP, DEX, and REM and 2.5 μg mL^−1^ for IS (DAC). These previous steps were repeated for the preparation of pre-extracted, post-extracted, and solvent samples of the 300 ng mL^−1^ ternary human samples.

After filtering each prepared sample using a membrane filter (pore size of 0.22 μm), a volume of 10 μL was analyzed under the suggested chromatographic conditions. The peak areas were obtained, and the matrix effect, extraction efficiency, process efficiency, and their IS normalized values were estimated for each group.

#### Experimental design

The purpose of experimental design, also known as design of experimentation (DOE), is to collect as much data as possible from as few experimental trials as possible so that statistical models can be developed and significant conclusions are derived^38^. The full factorial design with/without one centerpoint, response optimizer, and optimization plot were studied in this work^38,39^.

## Results and discussion

### Method development

#### Selection of column and guard column

According to a previous study^40^, the UPLC Ethylene (-CH_2_-CH_2_-) Bridged Hybrid (BEH) C_18_ columns were the first choice for UPLC separations^41^. A UPLC column BEH C_18_ 7 μm (2.1 $\times$ 100.0 mm) connected to the ultra-low volume guard column (2.1 $\text{x}$ 5-mm-length) efficiently maintains UPLC column performance^42^.

#### Selection of the mobile phase

According to a previous study^40^, methanol (HPLC grade, ≥ 99.9%) was chosen as an eco-friendly organic mobile phase (pH 7–8.3^43^), while ammonium acetate solution was chosen as the aqueous mobile phase. Ammonium acetate is commercially available at a low price. It is also a safe, chemically stable, and effective buffering medium with very high solubility in methanol. Ammonium acetate solution greatly enhances drug separations because of its excellent residual silanol masking effect on the chromatographic media, and it is harmonious with all LC detectors^44^.

#### Selection of the suitable wavelength

The PDA detector was theoretically required to be tuned above 215 nm because the UV cutoffs of both ammonium acetate and methanol are at 205 nm^45,46^. After the PDA scan of the tested drugs, 220 nm was set for OSTP, 315 nm was set for DAC (IS) (λ_max_ of DAC = 315 nm), and 245 nm was set for DEX (λ_max_ of DEX = 239 nm) and REM (λ_max_ of REM = 245 nm) (Fig. S2 and Table S2)^47^. As a result, PDA was set at three wavelengths (λ) during the in vivo analysis, which gave the highest responses for the tested drugs and hence the highest sensitivities. On the other hand, 239 nm was chosen during method development and factorial design optimization for all drug detections.

#### The drug order of separation

The separation sequence of the compounds is dependent on their log P and pka values^48^. Log P (Table S3) suggests that these medications should ideally elute in the following order: OSTP, DEX, and REM. However, the retention time and tailing of the chromatographic peak of a drug are significantly influenced by the pH of the mobile phase, the drug ionization, and the interaction of the ionized drug molecules with the free silanols within the stationary phase. So, based on their pKa values (Table S3), DEX and REM would not be greatly affected by the fluctuation of pH from 8 to 2, as they would be unionized. At the same time, OSTP retention time would be greatly affected by the pH of the mobile phase, as it would be less ionized at high pHs than at low values. So, at pH 7, the predicted order would be DEX, OSTP, and then REM. At pH 4, however, with effectively masked silanol groups, the expected order is OSTP, DEX, and finally REM. This is due to the fact that ionizable compounds have significantly shorter retention times than un-ionizable ones in RP- HPLC^48^.

#### Chromatographic trials during method development

It was suggested to choose favipiravir (FAV)^49^, DAC^50^, or ledipasivir (LED)^51^ as the internal standard (IS). Several conditions were tried during the development of the UPLC method (Table S2, Trials 1–35). DAC as an internal standard was chosen (Table S2 notes). It was observed that the retention times of OSTP and DAC were significantly influenced by pH of the ammonium acetate aqueous mobile phase. So, it was concluded that ammonium acetate (10–50 mM) as an aqueous mobile phase solution adjusted at pH 4 with phosphoric acid showed the most suitable peak order (OSTP, DEX, DAC, and then REM) with good system suitability parameters. Finally, Trials 35–38 (Table S2) were established to determine the independent factors of the factorial design and their levels.

### Method optimization by factorial design

#### Full factorial design with centerpoint experiment

Three independent factors affected the chromatographic performance: methanol percentage (MOH%) in the mobile phase at levels of 60 and 65%, flow rate (FR) at levels of 0.2 and 0.3 mL min^−1^, and ammonium acetate (AA) at levels of 10 and 50 mM (Table S2, Trials 35–38, and Table S4). The dependent responses obtained from the chromatograms at a UV wavelength of 239 nm were computerized. According to Table S4, *k*' _(OSTP)_ and R_S2 (DAC)_ as dependent responses were greatly affected by the selected independent factors (predictor variables). To determine the curvature possibility (lack of fit) of the selected dependent responses, 2^3^ full factorial designs with one center point experiment (total experiments = 9) (Table S4 and Fig. S3) were first done, and then multiple regression analysis was estimated.

The results of *k*' _(OSTP)_ and R_S2(DAC)_ obtained from the nine factorial designs were within the (0.81–1.30) and (1.50–4.56) ranges, respectively. Theoretically, the acceptable limit for *k*' is more than 0.5^52^, while the acceptable limit for R_S_ is more than 1.5^53^. However, the acceptable minimum limit in plasma analysis for *k*' is 1 and R_S_ is 2 at 25 °C.

#### Multiple regression analysis

From the data obtained from the full factorial with center-point designs, a three-predictor model using multiple regression analyses for *k*' _(OSTP)_ and R_S2 (DAC)_ was evaluated.

The obtained regression equations were:

1 $${k{\prime} }_{(\text{OSTP})}= 5.16710-\left(0.06495\times \text{MOH\%}\right)-\left(0.62680\times \text{FR}\right)+(0.00269\times \text{AA concentration})$$

2 $${\text{R}}_{\text{S}2 (\text{DAC})}= 25.64900-\left(0.33349\times \text{MOH\%}\right)-\left(2.92800\times \text{FR}\right)-(0.03133\times \text{AA concentration})$$

##### Interpreting the P-value for intercept

The intercept term in the regression (Table S5) indicates the average expected value for the dependent factor when all the independent factors are equal to zero.

From Eqs. (1) and (2) and from Table S5, the regression coefficients for the intercepts are equal to 5.16710 and 25.64900 for *k*' _(OSTP)_ and R_S2 (DAC)_, respectively. This means that at chromatographic conditions of zero methanol percentage, 0.00 mL min^-1^ flow rate, and 0 mM ammonium acetate, the average expected *k*' _(OSTP)_ and R_S2 (DAC)_ values are 5.167 and 25.65, respectively. In conducting regression analyses, a p-value for each regression coefficient is estimated. From Table S5, the p-values are 0.000 (less than α-level (0.05)), which means that the intercept term is statistically different from zero.

##### Interpreting the P-value for methanol percentage predictor variable

From Eqs. (1) and (2) and from Table S5, methanol% is a predictor variable that ranges from 60 to 65%. The regression coefficients for methanol percentage are − 0.06495 and − 0.33349 for *k*' _(OSTP)_ and R_S2 (DAC)_, respectively. This means that, on average, each additional percentage of methanol is associated with a decrease of 0.06495 and 0.33349 values on *k*' _(OSTP)_ and R_S2 (DAC)_, respectively, assuming the predictor variables flow rate and ammonium acetate concentration are held constant.

From Table S5, the corresponding p-values are 0.000, which are statistically significant at an alpha level of 0.05. This means that the average change in *k*' _(OSTP)_ and R_S2 (DAC)_ for each additional percentage of methanol is statistically significantly different than zero (i.e., methanol percentage has a statistically significant relationship with the response variables *k*' _(OSTP)_ and R_S2 (DAC)_).

##### Interpreting the P-value for flow rate predictor variable

From Eqs. (1) and (2) and from Table S5, the flow rate is a predictor variable that ranges from 0.2 to 0.3 mL min^−1^. The regression coefficients for the flow rate are − 0.62680 and − 2.92800 for *k*' _(OSTP)_ and R_S2 (DAC)_, respectively. This means that, on average, each additional mL min^−1^ of the flow rate is associated with a decrease of 0.62680 and 2.92800 values on *k*' _(OSTP)_ and R_S2 (DAC)_, respectively, assuming the predictor variables methanol percentage and ammonium acetate concentration are held constant.

From Table S5, the corresponding p-values are more than 0.05, which is not statistically significant at an alpha level of 0.05. This means that the average changes in *k*' _(OSTP)_ and R_S2 (DAC)_ for each additional mL min^−1^ of the flow rate are not statistically significantly different from zero (i.e., the flow rate does not have a statistically significant relationship with *k*' _(OSTP)_ and R_S2 (DAC)_). This indicates that the effect of the flow rate on *k*' _(OSTP)_ and R_S2 (DAC)_ could have been due to random chance.

##### Interpreting the P-value for ammonium acetate concentration predictor variable

From Eqs. (1) and (2) and from Table S5, ammonium acetate concentration (mM) is a predictor variable that ranges from 10 to 50 mM. The regression coefficients for ammonium acetate concentration (mM) are 0.00269 and − 0.03133 for *k*' _(OSTP)_ and R_S2 (DAC)_, respectively. This means that, on average, each additional mM of ammonium acetate is associated with an increase of 0.00269 and a decrease of 0.03133 values on *k*' _(OSTP)_ and R_S2 (DAC)_, respectively, assuming the predictor variables methanol percentage and flow rate are held constant.

From Table S5, the corresponding p-values are less than 0.05, which is statistically significant at an alpha level of 0.05. This means that the average change in *k*' _(OSTP)_ and R_S2 (DAC)_ for each additional mM of ammonium acetate is statistically significantly different than zero (i.e., ammonium acetate concentration (mM) has a statistically significant relationship with the response variables *k*' _(OSTP)_ and R_S2 (DAC)_).

From the previous interpretations of p-values, the optimized regression analyses for *k*' _(OSTP)_ and R_S2 (DAC)_ after removing the insignificant term (FR) from regression Eqs. (1) and (2) were evaluated to obtain Eqs. (3) and (4):

3 $${k{\prime} }_{(\text{OSTP})}= 5.01040-\left( 0.06495\times \text{MOH\%}\right)+(0.00269\times \text{AA concentration})$$

4 $${\text{R}}_{\text{S}2 (\text{DAC})}= 24.91700-\left(0.33349\times \text{MOH\%}\right)-(0.03133\times \text{AA concentration})$$

##### Interpreting the data subsetting lack of fit test, the R^2^, predicted R^2^, and adjusted R^2^

Minitab calculates two types of lack of fit tests. The first test is the pure error lack of fit test. This test is used if the data contains replicates and the model needs to be reduced. Replicates represent "pure error" because only random variation can cause differences between the observed response values. If we are reducing our model and the resulting p-value for lack of fit is less than 0.05, then we should retain the term we removed from the model. The second test is the data subsetting lack of fit test. This test is used if the data does not contain replicates and we want to determine if we are accurately modeling the curvature. This method identifies curvature in the data and interactions among predictors that may affect the model fit. Whenever the data subsetting p-value is less than 0.05, Minitab displays the message "Possible curvature in variable X." If evidence exists that this curvature is not adequately modeled, a higher-order term to model the curvature may be tried after examining the raw data in a scatter plot.

According to Table S5, the data subsetting lack of fit test was done. There was no evidence of curvature in the regression lines of the methanol percentage, flow rate, or ammonium acetate concentration against *k*' _(OSTP)_ or R_S2 (DAC)_, indicating that the optimum condition can be obtained from the 2^3^ full factorial design model without conducting a centerpoint experiment.

From Table S5, the R^2^ values indicate that the independent responses explain 96.4% and 96.8% of the variance in *k*' _(OSTP)_ and R_S2 (DAC)_, respectively. The adjusted R^2^ values were 94.3% and 95.0% for *k*' _(OSTP)_ and R_S2 (DAC)_, respectively, which accounts for the number of independent responses in the factorial design model. Both values indicate that the model fits the data well. The predicted R^2^ values were 90.50% and 91.28% for *k*' _(OSTP)_ and R_S2 (DAC)_, respectively. Because the predicted R^2^ values are close to the R^2^ and adjusted R^2^ values, the model does not appear to be overfit and has adequate predictive ability.

##### Interpreting the residual plots

**Fig. 1:**
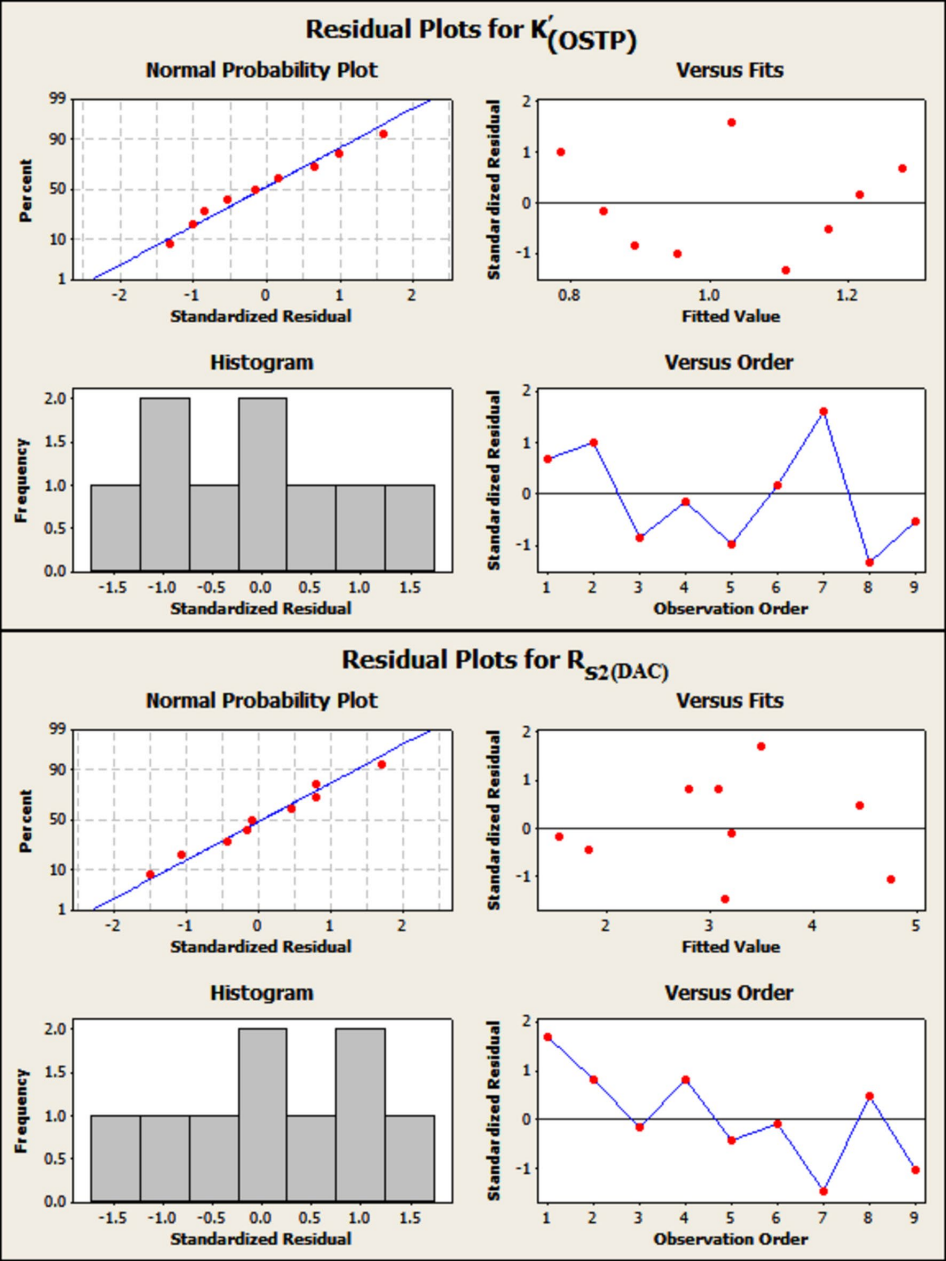
Residual plots for *k*' _(OSTP)_ and R_S2 (DAC)_.

The residual value is the difference between an observed value and its corresponding fitted value. The probability plot is used to evaluate the fit of a distribution to the data. The residual normal probability plots of *k*' _(OSTP)_ and R_S2 (DAC)_ show an approximately linear pattern consistent with a normal distribution (Fig. 1). The histogram is a graph used to assess the shape and spread of continuous sample data. The histogram can be created prior to or in conjunction with an analysis to help confirm assumptions and guide further analysis. To draw a histogram, minitab divides sample values into many intervals called bins. By default, bars represent the number of observations falling within each bin. According to Fig. 1, the residual histogram of *k*' _(OSTP)_ demonstrated the presence of three negative residual observations (less than − 0.25), four positive residual observations (more than 0.25), and three residual values between − 0.25 and 0.25. On the other hand, the residual histogram of R_S2 (DAC)_ demonstrated the presence of four negative residual observations (less than − 0.25), three positive residual observations (more than 0.25), and three residual values between − 0.25 and 0.25.

In addition, the residual plot histograms of *k*' _(OSTP)_ and R_S2 (DAC)_ indicate that there are no outliers (unusual large or small observations) that may exist in the data, as shown by the continuous bars on the standardized residuals axis of the plot. The plots of residuals versus the fitted values (predicted values) of *k*' _(OSTP)_ and R_S2 (DAC)_ show a random pattern of residuals on both sides of 0 (reference line) (Fig. 1). The plot of residuals versus the run order is a plot of all residuals in the order that the data was collected in Table S4 and can be used to find non-random errors. The residuals versus order plots of *k*' _(OSTP)_ and R_S2 (DAC)_ display a random pattern, so the residuals are uncorrelated with each other (Fig. 1).

#### *Optimum conditions determination by 2*^*3*^* FFD without center-point experiment*

Using a 2^3^ full factorial design (total experiments = 8), the response optimizer program was fed with the results of *k*' _(OSTP)_ and R_S2 (DAC)_ dependent responses with their desired lower, target, and upper values (Table S1). After program data analysis, an optimization plots (Table S1) was obtained and the optimum *k*' _(OSTP)_ and R_S2 (DAC)_ conditions with their desirability values and the highest composite desirability (D) value were evaluated^39^. According to Table S1, the final optimal UPLC conditions were 61.5% (v/v) methanol with 38.5% (v/v) AA (40 mM) as a mobile phase with a flow rate of 0.25 mL min^-1^ at a column oven temperature of 25 °C while adjusting the sample injection volume at 10 μL (Table S2, Trial 39).

#### Effect of column oven temperature

**Fig. 2:**
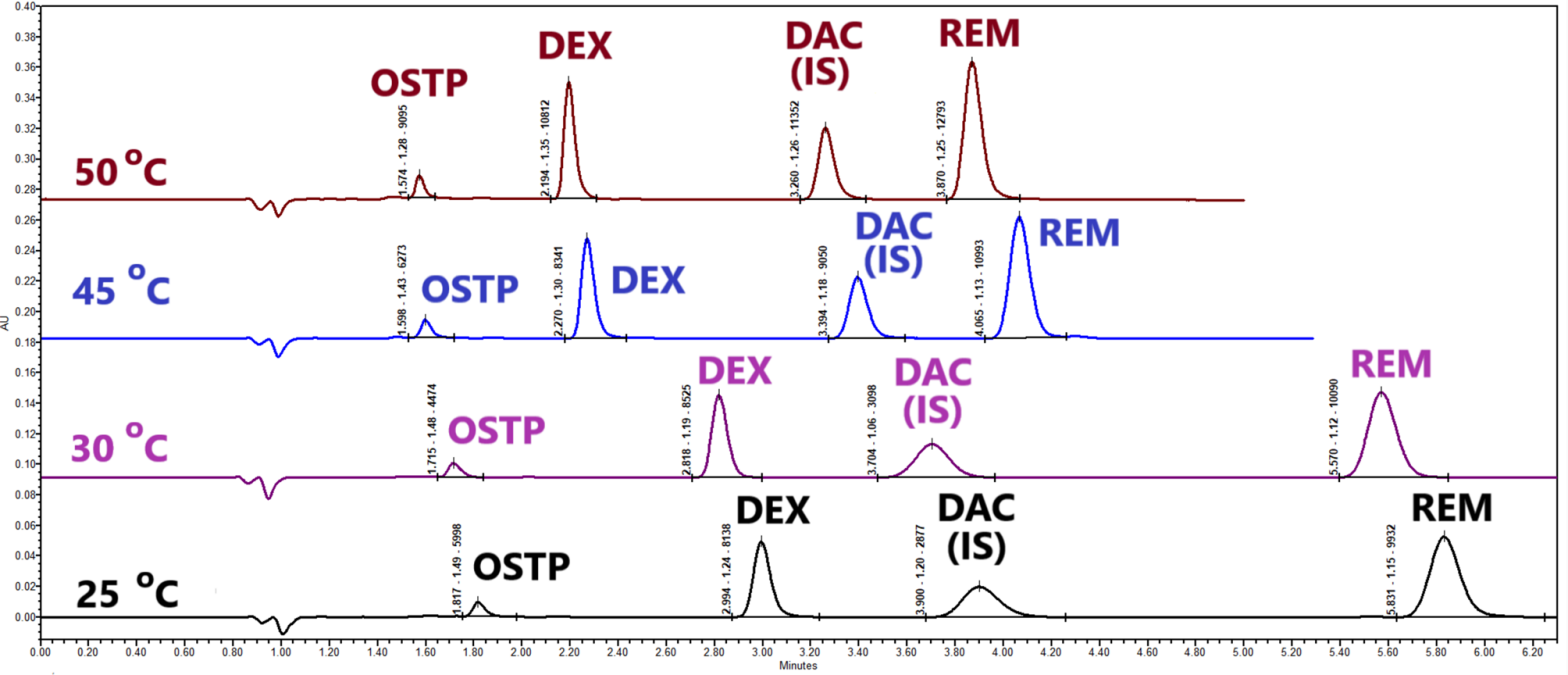
UPLC Chromatograms of oseltamivir phosphate, dexamethasone, daclatasvir dihydrochrolide (IS), and remdesivir (500 ng mL^−1^ each) were simultaneously separated at different column champer’s temperatures (25, 30, 45, and 50 °C) (each peak labeled by (Rt-T-N)).

UPLC column BEH C_18_ 1.7 μm (2.1 $\times$ 100 mm) can withstand elevated temperatures to 80 °C at low pH. Elevating the temperature of the column oven has many benefits. As the column oven’s temperature increases, the mobile phase’s viscosity decreases, and the column’s backpressure decreases. It was observed that raising the temperature from 25 to 50 °C reduced the column’s backpressure from 13,000 psi to 9000 psi. This enables higher flow rates to be used if needed. In addition, the lower pressure reduces the gradual damage to the UPLC instrumentation and thereby increases column life. Increased temperature improves column efficiency while decreasing operating pressure^54^. Also, as the temperature of the column oven is increased, the retention times of the peaks decrease (Fig. 2, Table 1, and Table S2, Trials 39–41).

**Table 1:** Percentage increase ( +) or decrease ( −) in retention times (Rt), capacity factor (K’), resolution (R), peak height (H), peak width (W), and number of theoretical plates (N) during increasing column oven temperatures from 25 °C to 50 °C.

Drug		OSTP			DEX			DAC (IS)			REM		
Column oven temperature		30 °C	45 °C	50 °C	30 °C	45 °C	50 °C	30 °C	45 °C	50 °C	30 °C	45 °C	50 °C
Percentage increase ( +) or decrease (-)^a^	Rt	− 5.6	− 12.1	** − 13.4**	− 5.9	− 24.2	** − 26.7**	− 5.0	− 13.0	** − 16.4**	− 4.5	− 30.3	** − 33.6**
	*K*’	− 6.2	− 28.0	** − 23.2**	‒	‒	**‒**	‒	‒	**‒**	‒	‒	**‒**
	R	‒	‒	**‒**	− 4.5	− 28.3	** − 21.6**	7.0	117.0	**140.4**	4.3	− 38.7	** − 36.2**
	Peak height (H)	− 4.8	+ 24.0	** + 56.0**	+ 9.1	+ 31.7	** + 54.5**	+ 9.4	+ 100.6	** + 133.1**	+ 6.1	+ 50.0	** + 70.3**
	Peak width (W)	− 15.8	− 14.4	** − 36.9**	− 19.7	− 28.3	** − 44.4**	− 16.5	− 45.2	** − 58.9**	− 26.8	− 45.0	** − 50.8**
	N	− 25.4	+ 4.6	** + 45.4**	+ 4.7	+ 2.5	** + 32.4**	+ 7.7	+ 214.6	** + 297.7**	+ 1.6	+ 10.7	** + 28.7**

Significant values are in bold.

^a^Percentage increase ( +) or decrease (-) from Rt, *K*’, R, H, W, and N values obtained at a column chamber temperature of 25 °C.

Chromatography is a sequence of equilibrium reactions in which the analytes are either dissolved in the mobile phase or adsorbed to the column’s stationary phase. The higher the temperature, the faster the analytes exchange between the mobile and stationary phases. The overlay of the chromatograms at 25, 30, 45, and 50 °C (Fig. 2 and Table 1) reveals that peak heights are increasing while peak widths are decreasing. As a result, the number of theoretical plates (N) of the peaks and the sensitivity of the suggested method increase.

The effect of the column oven’s temperature on retention times in reversed-phase chromatography is largely determined by the Gibbs free energy (ΔG°) of the solute interaction with the stationary phase. The ΔG° energy is estimated from the slope of plots of log *K*' versus 1/T, called van't Hoff plots (Eq. (5)).

5 $$\text{ln}{K}{\prime}= - \frac{\Delta {\text{G}}^{^\circ }}{\text{RT}}+\text{ln}\Phi $$

where *K*' is the capacity factor, R is the gas constant (8.3145 J mol^−1^K^−1^), T is the absolute temperature in kelvin, and Φ is the phase ratio of the column^55–57^. The phase ratio is defined as the ratio between the volume of the stationary phase (V_s_) and the volume of the mobile phase (void volume, V_o_) in a column^58^.

**Fig. 3:**
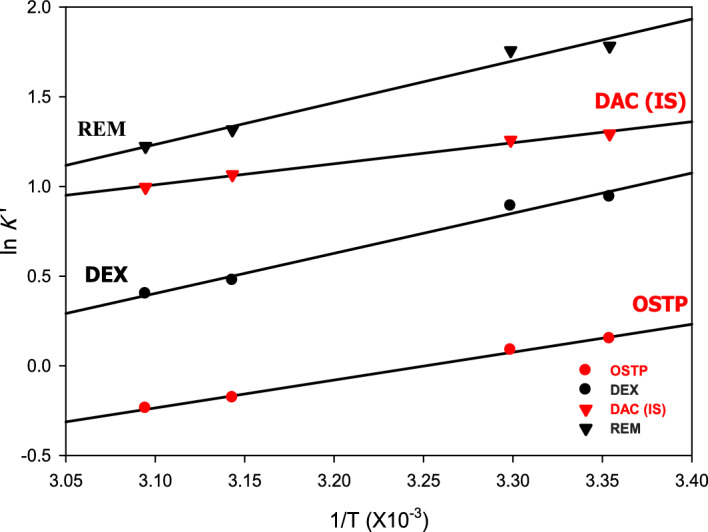
Van’t Hoff plots of oseltamivir phosphate, dexamethasone, daclatasvir dihydrochrolide (IS), and remdesivir.

Van't Hoff plots were constructed for each drug (Fig. 3). In general, for components with identical stationary phase interactions, the Van't Hoff plots are linear with slightly different slopes as a result of minor differences in ΔG° values. If Van't Hoff plots deviate from linearity, the retention of the solute molecules in the stationary phase results from very different types of interactions, and the temperature in this case had a great influence on the selectivity^57^.

**Fig. 4:**
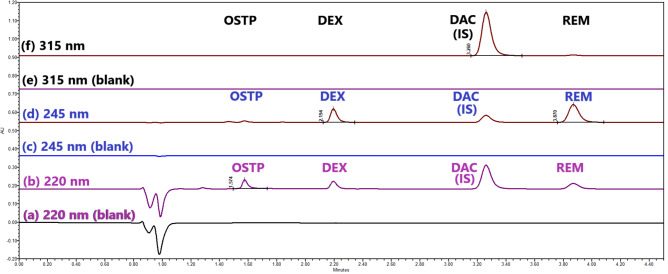
Chromatograms of 30% (v/v) aqueous methanol (blank), oseltamivir phosphate, dexamethasone, daclatasvir dihydrochrolide (IS), and remdesivir (500 ng mL^−1^ each) at different PDA channels (220, 245, and 315 nm) and column temperature of 50 °C.

From Table 2 and Fig. 3, it was observed that OSTP and DAC (IS) had identical stationary phase interactions as their Van't Hoff plots had slightly different slopes and minor differences in ΔG° values, as well as for DEX and REM. Furthermore, Van't Hoff plots for OSTP and DAC (R^2^ greater than 0.99) were linear, while those for DEX and REM deviated slightly from linearity (R^2^ lower than 0.99), which indicated differences in the retention mechanisms. The column oven temperature of 50 °C was the most suitable temperature according to Table 1, with good system suitability parameters (Fig. 4 and Table S2, Trial 42).

**Table 2:** Gibbs’ free energy (ΔG, KJ mol^−1^) of each drug’s interaction with the stationary phase.

Drug	Coefficient of determination (R^2^)	Slope (b) = $- \frac{\Delta {\text{G}}^{\text{o}}}{\text{R}}$	ΔG (KJ mol^−1^)
OSTP	0.9963	1.55 × 10^3^	− 0.19
DEX	0.9854	2.24 × 10^3^	− 0.27
DAC (IS)	0.9916	1.17 × 10^3^	− 0.14
REM	0.9770	2.33 × 10^3^	− 0.28

#### The drug extraction technique from human plasma

OSTP, DEX, DAC (IS), and REM have high plasma protein binding of 42%^59^, 77%^60^, 99%^50^, and 88–93.6%^7^, respectively. They also have high log Ps of 1.30^59^, 1.93^60^, 3.47^50^, and 2.10–3.20^7^, respectively. As a result, the most suitable and available method of extraction was liquid–liquid extraction (LLE). Unlike plasma protein precipitation techniques, LLE does not precipitate plasma proteins with the attached drugs. As a result, the drug recovery from plasma by LLE is greater than that achieved by protein precipitation techniques. Also, the high log Ps of the studied drugs permit good extraction of these nonpolar drugs into the organic phase. LLE allows the purification and pre-concentration of the evaporated residues in a small amount of the appropriate solvent, which increases the sensitivity of the method to detect small amounts of the studied drugs in plasma. So, this technique is suitable for drugs with a low plasma C_max_.

LLE is a separation technique that is frequently employed in industrial operations as well as on the laboratory scale due to its simplicity, low cost, and applicability for thermally labile and high-boiling chemicals^61^. However, the main disadvantages of LLE are the consumption of high volumes of organic solvents needed for the extraction of the drug, a time-consuming process when compared to other methods, the requirement of an evaporation step prior to analysis to remove excess organic solvent, and the possibility of emulsion formation when two immiscible phases are used in the extraction procedure. To overcome these disadvantages, one organic solvent was used to ease the extraction process. A low volume of 2 mL of the organic solvent per sample was used to minimize the pollution effect. Diethyl ether (DEE) as an extraction solvent was used. It is most commonly used in LLE. It has high volatility properties at low temperatures (boiling point: 34.6 °C). So, it is suitable for the extraction of thermolabile drugs. DEE is an inert compound with a high solvation capacity for nonpolar compounds because ethers do not have a hydrogen bonding network that would have to be broken up to dissolve the solute^62^.

#### Drugs extraction from human blood withdrawn in different types of blood withdrawing tubes

Samples of human blood were withdrawn in different tubes (K_3_EDTA, fluoride, and fluoride-EDTA) to study their effect on the stability of the studied drugs. The same procedure under Section “assessment of bench-top (short-term) stability” was adopted for this study. Under the final chromatographic conditions of Table 3, the stability of the tested drugs in these kits was studied at time intervals of 0, 1, and 2 h (Eq. (6) and Table S6).

**Table 3:** Optimum chromatographic conditions for the RP-UPLC-PDA separation of the oseltamivir phosphate/dexamethasone/remdesivir mixture in human plasma.

Column	UPLC column BEH C_18_ 1.7 μm (2.1 × 100 mm) connected with UPLC guard-column BEH 1.7 μm (2.1 × 5 mm)
Mobile phase	Methanol : Ammonium acetate (40 mM) adjusted to pH 4 = 61.5:38.5 (v/v)
Full run time	5 min
Flow rate	0.25 mL min^−1^
Temperature	50 °C at column oven and 25 °C at autoinjector part
Injection volume	10 µL
PDA detector	220 nm for OSTP, 315 nm for IS, and 245 nm for DEX and REM

6 $$\text{Stability at }(\text{x})\text{ hours }= \frac{\frac{{\text{AUC}}_{\text{Drug}}}{{\text{AUC}}_{\text{IS}}}\text{ at }(\text{x})\text{ hours}}{\frac{{\text{AUC}}_{\text{Drug}}}{{\text{AUC}}_{\text{IS}}}\text{ at }(0)\text{ hours}} \times 100$$

Where AUC is the area under the curve response obtained from the chromatogram.

It was observed that fluoride-EDTA plasma deactivated the esterase enzyme of human blood plasma more than EDTA or fluoride alone and hence increased the stability of the analyzed drugs, especially OSTP^63–65^ (Table S6).

### Method characteristics

A methanolic quaternary mixture of 500 ng mL^-1^ of each drug was prepared to determine the independent factors. The 2^3^ full factorial designs were applied at a column oven temperature of 25 °C. The effect of the temperature of the column oven on drugs’ separation was studied. Finally, the optimum conditions of the proposed method were developed for simultaneous determination of OSTP, DEX, and REM in human plasma using DAC as the IS (Table 3).

We confirm that all experiments were performed in accordance with relevant guidelines and regulations. The study was conducted according to the rules of the Ethics Committee at the Faculty of Pharmacy, Suez Canal University (number: 202009PHDH1). Informed consent was obtained from all subjects and/or their legal guardian(s).

### Method validation

The proposed method was validated according to the bioanalytical method validation M10 ICH^31^, FDA^32^, and EMA’s^33^ guidelines. The validation parameters of linearity, limits of detection and quantitation, accuracy and precision, selectivity and specificity, system suitability parameters, stability of stock solutions, freeze–thaw cycles, short-term, long-term, and processed treated samples stability tests, matrix effect, extraction efficiency (recovery), and process efficiency and their IS normalized values were evaluated:

#### Linearity

Six concentrations were analyzed to evaluate the method's linearity for each drug (Table 4). The suggested method's calibration curves (n = 6) (Fig. S4) were plotted within the stated linearity limits for each drug, as shown in Table S7. The high coefficients of determination (R^2^ > 0.9930) suggested that the calibration curves were linear. Table 4 summarizes the quantitative statistical criteria for the studied drugs.

**Table 4:** Statistical parameters of calibration curves of oseltamivir phosphate/dexamethasone/remdesivir in human plasma using the developed method (n = 6).

Parameter	Oseltamivir phosphate	Dexamethasone		Remdesivir	
Linearity range (ng mL^−1^)	200–5000	10–500	500–5000	20–500	500–5000
Intercept (a)	− 0.029	− 0.002	− 0.038	0.274	0.490
Slope (b)	$1.56\times {10}^{-4}$	$11.69 \times {10}^{-4}$	$15.15 \times {10}^{-4}$	$25.37 \times {10}^{-4}$	$24.46 \times {10}^{-4}$
Coefficient of determination (R^2^)	0.9999	0.9996	0.9942	0.9980	0.9980
SD of intercept (Sa)	0.001	0.003	0.132	0.015	0.135
SD of slope (Sb)	$5.85\times {10}^{-7}$	$120.60\times {10}^{-7}$	$579.91\times {10}^{-7}$	$572.49\times {10}^{-7}$	$590.66\times {10}^{-7}$
SD of residuals (Sy/x)	0.002	0.005	0.224	0.023	0.228
Mean recovery ± SD, %	101.169 ± 3.856	101.613 ± 6.401	97.742 ± 8.905	100.877 ± 4.796	99.172 ± 9.927
RSD, %^a^	3.812	6.300	9.111	4.754	10.010
Error, %^b^	1.574	2.613	3.636	1.958	4.053

^a^Percentage relative standard deviation for six samples.

^b^Percentage relative error for six samples.

These linearity ranges cover their concentrations in human plasma. Oral doses of 500- and 1000-mg OSTP have a C_max_ of 564 (± 222) and 809 (± 216) ng mL^−1^, respectively^66^. The plasma C_max_ of oral 1.5-mg DEX, intramuscular 3-mg DEX injection, and bolus intravenous 4-mg DEX injection are 13.9 ± 6.8^67^, 34.6 ± 6.0^67^, and nearly 100 ng mL^−1^^68^, respectively. In high single doses of DEX in cancer patients, the plasma C_max_ reaches 1000‒5000 ng mL^−1^^69,70^. The intravenous infusion of 3-mg REM for two hours yields a plasma mean C_max_ of 57.5 ng mL^−1^^71^, while the intravenous infusion of 225-mg REM for two hours yields a plasma mean C_max_ of 4420 ng mL^−1^^71^.

#### Accuracy and precision

The suggested method's accuracy and precision for the within runs (n = 5) and for the between runs within three days (n = 3 per day) were evaluated using low (LQC), middle (MQC), and high (HQC) quality controls at each linearity range besides their lower limits of quantitation (LLOQ) (Table S8). The standard deviation (SD) and percentage relative standard deviation (% CV) of the obtained findings were calculated. The CV was found to be small (less than 8.31% for OSTP, 11.41% for DEX, and 11.01% for REM), indicating that the proposed method had appropriate accuracy and precision (Table S8).

#### Selectivity and specificity

**Fig. 5:**
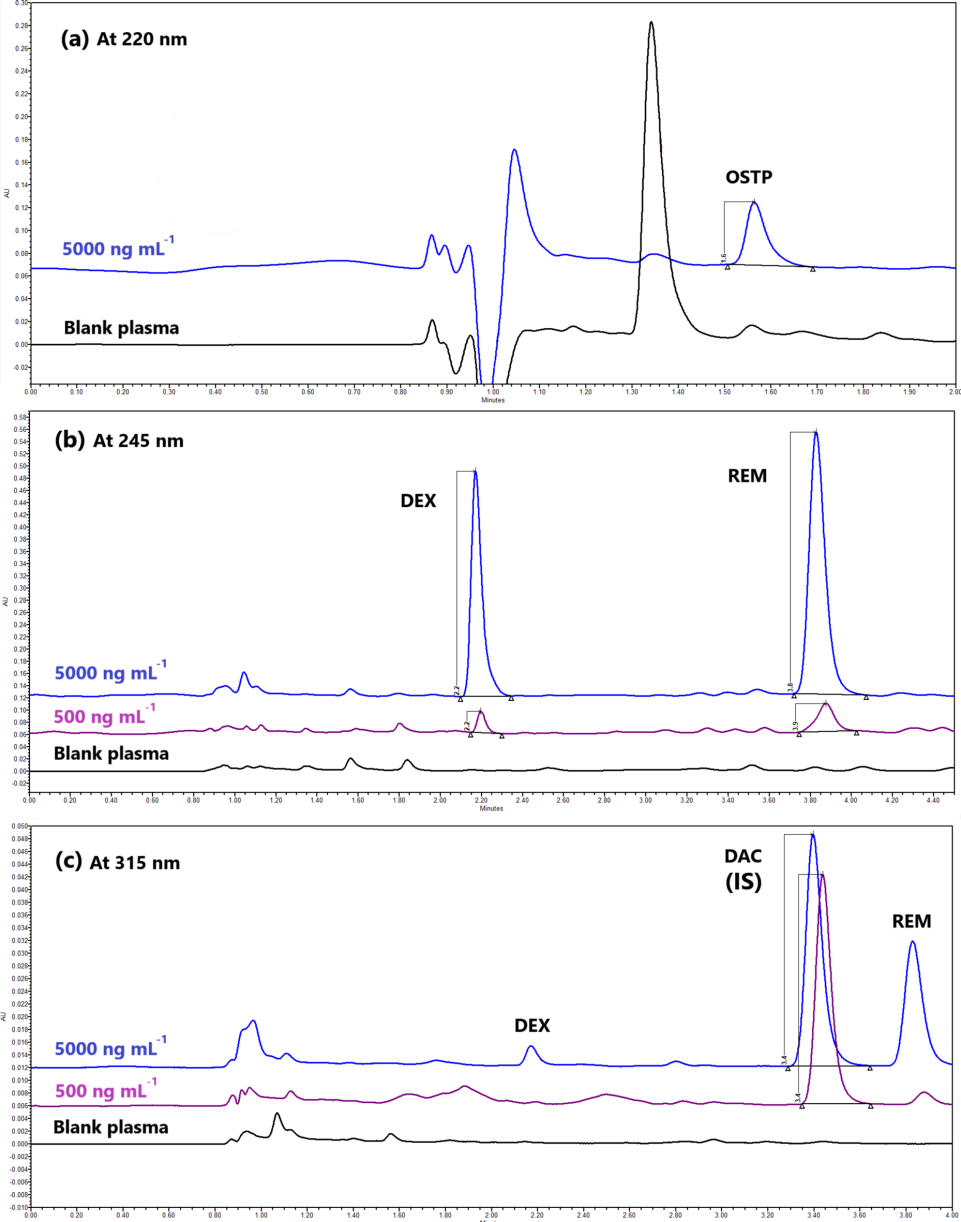
Chromatograms of plasma extracted by the proposed method for specificity.

Blank, OSTP, DEX, DAC (IS), and REM in human plasma were all investigated at different wavelengths. The blank chromatograms illustrated no peaks within the retention times of the studied drugs. Moreover, no peaks were noticed within any of the drug chromatograms except for their own. So, they were prepared in a quaternary solution (Fig. 5). The retention times of OSTP, DEX, the internal standard (DAC), and REM were 1.563 ± 0.006, 2.178 ± 0.017, 3.416 ± 0.016, and 3.826 ± 0.021 min (eight replicates), respectively.

#### System suitability parameters

The system suitability parameters were determined by using “Equations (S1-S4)”^39^. According to Table 5, the RSD% of system suitability parameters obtained from eight chromatograms on four separate days suggested that the system was performing effectively.

**Table 5:** System suitability parameters for RP-UPLC-PDA determination of oseltamivir phosphate/dexamethasone/daclatasvir dihydrochloride/remdesivir mixture in plasma.

Parameter	Oseltamivir phosphateat 220 nm	Dexamethasone at245 nm	Daclatasvirdihydrochloride (IS) at 315 nm	Remdesivir at245 nm
R_t_ (mean ± SD^a^, RSD%)^b^	1.563 ± 0.006, 0.376%	2.178 ± 0.017, 0.766%	3.416 ± 0.016, 0.469%	3.826 ± 0.021, 0.555%
*k*'^c^ (mean ± SD, RSD%)	0.784 ± 0.012, 1.578%	‒	‒	‒
R_S_^d^ (mean ± SD, RSD%)	‒	7.557 ± 0.504, 6.663%	10.814 ± 1.062, 9.824%	4.096 ± 0.658, 16.057%
T^e^ (mean ± SD, RSD%)	1.023 ± 0.120, 11.724%	1.547 ± 0.127, 8.204%	1.304 ± 0.011, 0.824%	1.243 ± 0.021, 1.691%
N^f^ (mean ± SD, RSD%)	12,856.391 ± 2066.184,16.071%	10,077.492. ± 1404.036,13.932%	11,575.463 ± 835.778, 7.220%	12,227.516 ± 855.264, 6.995%

^a^Standard deviation (4–8 replicates at three different days).

^b^Percentage relative standard deviation (4–8 replicates).

^c^The analyte peak's capacity factor.

^d^The resolution between two successive analytes’ peaks.

^e^The analyte peak's tailing factor.

^f^The number of theoretical plates of the analyte peak.

#### Stability

##### Stability of the analyte and IS in stock and working solutions

According to a previous study^40^, the stock and working solutions were stable in the freezer for nearly three weeks for OSTP and DEX analysis but for only about one week in the case of DAC and REM analysis.

##### The freeze–thaw cycles, short-term, long-term, and processed treated samples stability tests

The freeze–thaw cycles, the short-term stability, the long-term stability for one week, and the stability in processed treated samples for 300 and 1000 ng mL^-1^ of each drug were estimated (Table 6). The tested quality control samples showed high percent recoveries within the range of 86.72% to 106.23% of the nominal concentration, which lies within the acceptable range. The relative standard deviation percentage (RSD% or CV%) of precision was not more than 12.15% (Table 6).

**Table 6:** The stability data of oseltamivir phosphate/dexamethasone/remdesivir mixture in human plasma and processed treated samples under different storage conditions (n = 3).

Drug name		OSTP				DEX				REM			
Stability assessment test	Storage condition	300 ng mL^−1^		1000 ng mL^−1^		300 ng mL^−1^		1000 ng mL^−1^		300 ng mL^−1^		1000 ng mL^−1^	
		Accuracy^a^ (Mean, %)	Precision^b^ (CV, %)	Accuracy^a^ (Mean, %)	Precision^b^ (CV, %)	Accuracy^a^ (Mean, %)	Precision^b^ (CV, %)	Accuracy^a^ (Mean, %)	Precision^b^ (CV, %)	Accuracy^a^ (Mean, %)	Precision^b^ (CV, %)	Accuracy^a^ (Mean, %)	Precision^b^ (CV, %)
1- Freeze–thaw cycles	− 80 °C then 25 °C (3 cycles)	103.44	3.42	100.00	4.74	105.18	4.49	99.31	10.78	99.59	1.48	97.62	12.15
2- Short-term stability	Bench-top (25 °C) for 2 h	101.24	2.52	93.46	3.31	98.53	3.14	101.38	3.33	98.52	2.00	94.92	1.07
	Freezer (− 20 °C) for 24 h	96.29	4.31	99.71	0.83	100.73	0.69	106.23	3.53	106.06	0.32	104.70	8.27
3- Long-term stability at − 80 °C	For one week	87.84	2.15	99.85	1.56	98.41	10.92	102.72	2.91	104.45	1.18	100.82	1.57
4- Stability in processed treated samples at − 80 °C	For 24 h	86.72	6.35	100.58	2.37	99.86	7.10	94.77	0.63	99.58	2.47	90.38	6.06

^a^The acceptance criteria were ± 15% of the nominal concentration’s values for QC samples.

^b^The acceptance criteria of the coefficient of variation (CV, %) values should not exceed 15% for QC samples.

#### Matrix effect, extraction efficiency, and process efficiency

They were estimated at low and high levels of QC. The 300 (LQC) and 1000 (HQC) ng mL^-1^ of each drug were analyzed using blank human plasma from four different sources (plasma A, B, C, and D).For each analyzed drug, the matrix effect (%ME), extraction efficiency or extraction coefficient (%Recovery efficiency, %RE), and process efficiency (%PE) and their IS normalized values were calculated.

The matrix effect (%ME) is defined as an alteration of the analyte response due to an interfering component (s) in the sample matrix (human plasma) (Eq. (7)). The IS normalized matrix effect (%) is defined as the difference percentage in the "response of the analyte/response of IS" ratio between the same analyte concentrations in the sample matrix (human plasma) spiked with the analyte after extraction and in pure solvents (Eq. (8))^72^. The accuracies of %ME and the IS normalized matrix effect (%) should be within ± 15% of the nominal concentration, and the %RSD should not be greater than 15%^31^. As indicated in Table S9, the %RSD of %ME and IS normalized ME (%) values were not more than 2.37%, 5.02%, and 4.67% for OSTP, DEX, and REM, respectively, at both concentration levels. Thus, the plasma matrix did not appear to interfere significantly with the method.

The extraction efficiency (%recovery efficiency, %RE) is the percentage of solute that moves into the extracting phase (Eq. (9)). The high extraction efficiency percentage indicates high extraction process effectiveness, high method sensitivity, and high drug stability in the matrix. The IS normalized extraction efficiency (%) is defined as the difference percentage in the "response of the analyte/response of IS" ratio between the same analyte concentrations in the sample matrix (human plasma) spiked with the analyte before extraction and in the sample matrix (human plasma) spiked with the analyte after extraction (Eq. (10)). As indicated in Table S9, the %RE and IS normalized RE (%) values were more than 93%, 88%, and 94% for OSTP, DEX, and REM, respectively, at both concentration levels. Thus, the proposed method appeared to have high extraction efficiency for the tested drugs.

The process efficiency (%PE) is expressed as the ratio of the response of an analyte spiked in the sample matrix (human plasma) before extraction to the response of the same analyte in pure solvents multiplied by 100 (Eq. (11)). It would be equivalent to the recovery as per M10 ICH Guidelines^31^. The RE% value represented the true recovery value that was not affected by the sample matrix (human plasma), while the PE% value represented the overall process efficiency that was unfortunately affected by the sample matrix (human plasma)^73^. The IS normalized process efficiency (%) is defined as the difference percentage in the "response of the analyte/response of IS" ratio between the same analyte concentrations in the sample matrix (human plasma) spiked with the analyte before extraction and in pure solvents (Eq. (12)).As indicated in Table S9, the %RSD of %PE and IS normalized PE (%) values were not more than 5.04%, 5.08%, and 5.02% for OSTP, DEX, and REM, respectively, at both concentration levels. Thus, the proposed method appeared to have good overall process efficiency.

7 $$\text{Matrix effect }(\text{\%ME}) =\frac{{\text{AUC}}_{\text{Blank sample of plasma spiked with the drug after extraction }}}{{\text{AUC}}_{\text{Pure drug solution in }60\text{\% aqueous methanol }}} \times 100$$

8 $$\text{IS normalized matrix effect }(\text{\%}) =\frac{\left(\frac{{\text{AUC}}_{\text{Blank sample of plasma spiked with the drug after extraction }}}{{\text{AUC}}_{\text{Blank sample of plasma spiked with the IS after extraction }}}\right)}{\left(\frac{{\text{AUC}}_{\text{Pure drug solution in }60\text{\% aqueous methanol }}}{{\text{AUC}}_{\text{Pure IS solution in }60\text{\% aqueous methanol }}}\right)} \times 100= \frac{{\text{\%ME}}_{\text{Drug }}}{{\text{\%ME}}_{\text{IS }}} \times 100$$

9 $$\text{Extraction efficiency }(\text{\%Recovery efficiency},\text{ \%RE}) =\frac{{\text{AUC}}_{\text{Blank sample of plasma spiked with the drug before extraction }}}{{\text{AUC}}_{\text{Blank sample of plasma spiked with the drug after extraction }}} \times 100$$

10 $$\text{IS normalized extraction efficiency }(\text{\%}) =\frac{\left(\frac{{\text{AUC}}_{\text{Blank sample of plasma spiked with the drug before extraction }}}{{\text{AUC}}_{\text{Blank sample of plasma spiked with the IS before extraction }}}\right)}{\left(\frac{{\text{AUC}}_{\text{Blank sample of plasma spiked with the drug after extraction }}}{{\text{AUC}}_{\text{Blank sample of plasma spiked with the IS after extraction }}}\right)} \times 100= \frac{{\text{\%RE}}_{\text{Drug }}}{{\text{\%RE}}_{\text{IS }}} \times 100$$

11 $$\text{Process efficiency }(\text{\%PE}) =\frac{{\text{AUC}}_{\text{Blank sampleof plasma spiked with the drug before extraction }}}{{\text{AUC}}_{\text{Pure drug solution in }60\text{\% aqueous methanol }}} \times 100$$

12 $$\text{IS normalized process efficiency }(\text{\%}) =\frac{\left(\frac{{\text{AUC}}_{\text{Blank sample of plasma spiked with the drug before extraction }}}{{\text{AUC}}_{\text{Blank sample of plasma spiked with the IS before extraction }}}\right)}{\left(\frac{{\text{AUC}}_{\text{Pure drug solution in }60\text{\% aqueous methanol }}}{{\text{AUC}}_{\text{Pure IS solution in }60\text{\% aqueous methanol }}}\right)} \times 100=\frac{{\text{\%PE}}_{\text{Drug }}}{{\text{\%PE}}_{\text{IS }}} \times 100$$

### Greenness assessment

The greenness of the proposed method was assessed using the analytical Eco-Scale^35^, the Green Analytical Procedure Index (GAPI)^36^, and the AGREE method^34^. The analytical Eco-Scale^35^ is a semi-quantitative tool for greenness assessment of the analytical methods. The simultaneous analysis of OSTP, DEX, and REM in human plasma by the proposed method was an acceptable green analysis with low laboratory needs (Eco-score 68). Moreover, the suggested method provided acceptable green analysis when applied to the assessment of OSTP with DEX mixture, DEX with REM mixture, or each drug separately in human plasma (Eco-score greater than 50) (Table S10). Moreover, the suggested method provided acceptable green analysis when applied to the assessment of OSTP with DEX mixture, DEX with REM mixture, or each drug separately in human plasma (Eco-score greater than 50) (Table S10).

According to the Green Analytical Procedure Index (GAPI)^36^, the suggested method was an indirect procedure with a LLE extraction process (Fig. S5). It also used small volumes of safe chemicals with little waste due to the low flow rate (0.25 mL min^−1^). Furthermore, the suggested method was for qualitative and quantitative analyses.

The AGREE method (Analytical GREEnness Metric Approach and Software)^34^ is a comprehensive method that incorporates 12 significance principles for greenness assessment of the analytical methods. The obtained colorful pictogram shows the structure of weak and strong points of the analytical method. According to Fig. S6, the total score was illustrated in the center of the pictogram, with a number near one (0.62) and a light green color suggesting that the suggested method was acceptable green.

## Conclusion

The proposed method was optimized and validated according to the M10 ICH's bioanalytical method validation guideline. The developed method is highly sensitive, accurate, and precise, with a wide range of linearity in human plasma. It is suitable for assaying OSTP, DEX, and REM in human plasma, as the linearity ranges cover their plasma C_max_. The PDA detector is used for OSTP, DEX, and REM analysis using three wavelengths. The full factorial designs with one centerpoint experiment were used to determine the curvature possibilities (lack of fit) of the selected dependent responses, followed by multiple regression analysis. There was no evidence of curvatures in the regression lines of the methanol percentage, flow rate, or ammonium acetate concentration against *k*' _(OSTP)_ or R_S2 (DAC)_, indicating that the optimum condition can be obtained from the 2^3^ full factorial design without conducting a centerpoint experiment. The method was optimized at a column oven temperature of 25 °C. The effect of column oven temperatures at 25, 30, 45, and 50 °C on the drugs’ retention times was investigated by the Van't Hoff plots and Gibbs’ free energy (G°) of the tested drugs. OSTP and DAC (IS) have identical stationary phase interactions as their Van't Hoff plots had slightly different slopes and minor differences in ΔG° values, as well as for DEX and REM. The column oven temperature of 50 °C is the most suitable temperature with good system suitability parameters. The benchtop (short-term) stability of each drug at room temperature for 2 h in different types of human plasma was investigated. Fluoride-EDTA human plasma shows the highest stability results for the studied drugs. The matrix effect, extraction efficiency, process efficiency, and internal standard normalized values of the proposed method are high. The optimization by factorial method is the most eco-friendly technique. Because it consumes the least amount of instrumental energy and money and releases the least amount of hazardous waste into the environment. Furthermore, the analytical Eco-Scale, GAPI, and AGREE proved the greenness of the proposed method. It uses small volumes of safe chemicals with little waste due to the low flow rate (0.25 mL min^−1^).

## Supplementary Information

Supplementary Information.

## Data Availability

The datasets generated and/or analyzed during the current study are available from the corresponding author on reasonable request.
