# Supplementary material for: UPLC-PDA factorial design assisted method for simultaneous determination of oseltamivir, dexamethasone, and remdesivir in human plasma
Source: Sci Rep. 2024 Sep 18;14:21758. doi: 10.1038/s41598-024-71413-3 (PMC11411088; doi:10.1038/s41598-024-71413-3)
Supplement: Supplementary file 1 — Supplementary Information. [file 41598_2024_71413_MOESM1_ESM.pdf]

Supplementary materials

UPLC-PDA factorial design assisted method for simultaneous determination of oseltamivir, dexamethasone, and remdesivir in human plasma

Hanan I. EL-Shorbagy[*, 1], Mona A. Mohamed[2], Alaa El-Gindy[1], Ghada M. Hadad[1], Fathalla Belal[3].

[1] Pharmaceutical Analytical Chemistry Department, Faculty of Pharmacy, Suez Canal University, Ismailia 41522, Egypt.

[2] Pharmaceutical Chemistry Department, Egyptian Drug Authority (EDA), Cairo, Egypt.

[3] Department of Pharmaceutical Analytical Chemistry, Faculty of Pharmacy, Mansoura University, Mansoura 35516, Egypt.

* Corresponding author. PGS.202214@pharm.suez.edu.eg & Hananibrahimelshorbagy@gmail.com.

Table of supplementary material figure captions:

Fig. S1: Molecular structures and IUPAC names of (A) oseltamivir phosphate, (B) dexamethasone, and (C) remdesivir.

Fig. S2: PDA scans (200-400 nm) of oseltamivir phosphate, dexamethasone, daclatasvir dihydrochloride (internal standard), and remdesivir.

Fig. S3: The nine runs of 2^3 FFD experiments with centerpoint experiment of the analyzed drugs.

Fig. S4: Calibration curves of oseltamivir phosphate, dexamethasone, and remdesivir in human plasma.

Fig. S5: GAPI assessment of the green profile of the proposed method.

Fig. S6: Result of AGREE analysis for the proposed method.

Fig. S1

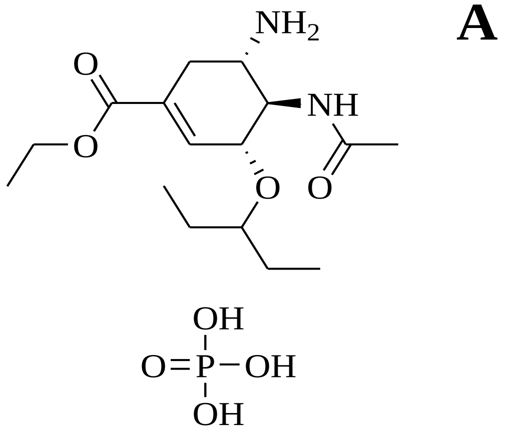

Ethyl (3R,4R,5S)-4-acetamido-5-amino-3-pentan-3-yloxycyclohexene-1-carboxylate;phosphoric acid

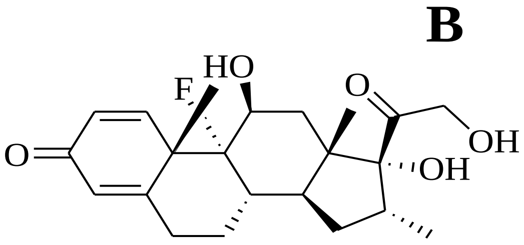

(8S,9R,10S,11S,13S,14S,16R,17R)-9-fluoro-11,17-dihydroxy-17-(2-hydroxyacetyl)-10,13,16-trimethyl-6,7,8,11,12,14,15,16-octahydrocyclopenta[a]phenanthren-3-one

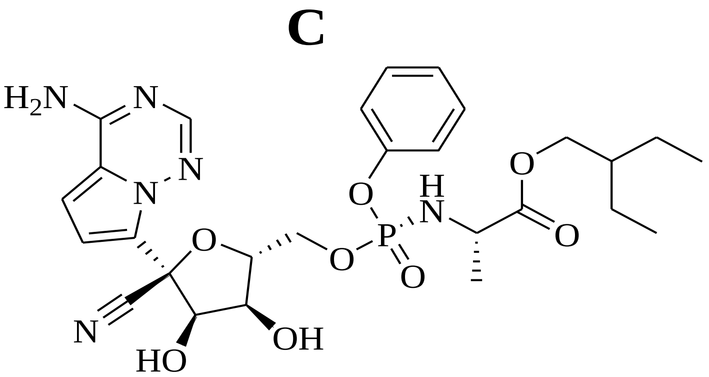

2-ethylbutyl (2S)-2-[[[(2R,3S,4R,5R)-5-(4-aminopyrrolo[2,1-f][1,2,4]triazin-7-yl)-5-cyano-3,4-dihydroxyoxolan-2-yl]methoxy-phenoxyphosphoryl]amino]propanoate

Fig. S2

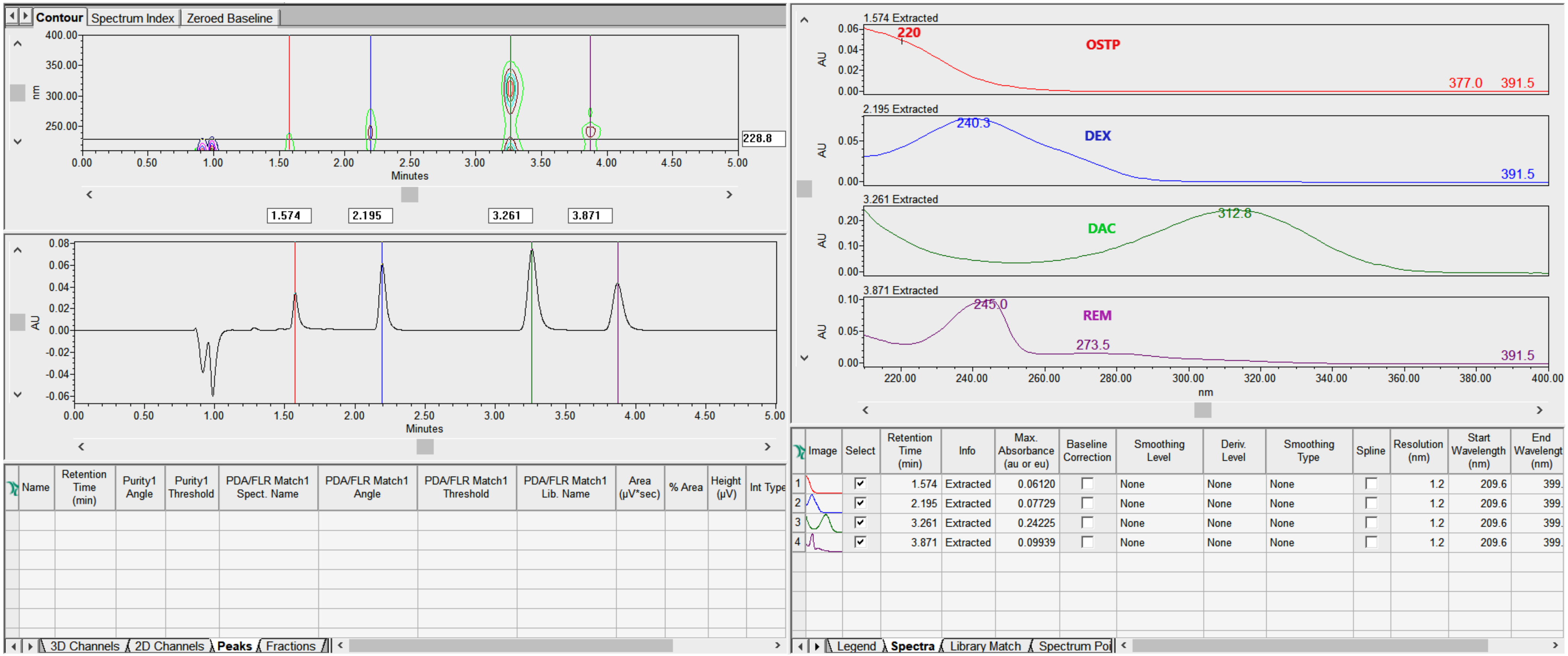

Contour
Spectrum Index
Zeroed Baseline
nm
Minutes
228.8
1.574
2.195
3.261
3.871
AU
1.574 Extracted
220
OSTP
377.0
391.5
2.195 Extracted
240.3
DEX
391.5
3.261 Extracted
312.8
DAC
3.871 Extracted
245.0
REM
273.5
391.5
nm
Name
Retention Time (min)
Purity1 Angle
Purity1 Threshold
PDA/FLR Match1 Spect. Name
PDA/FLR Match1 Angle
PDA/FLR Match1 Threshold
PDA/FLR Match1 Lib. Name
Area (µV*sec)
% Area
Height (µV)
Int Type
3D Channels
2D Channels
Peaks
Fractions
Image
Select
Retention Time (min)
Info
Max. Absorbance (au or eu)
Baseline Correction
Smoothing Level
Deriv. Level
Smoothing Type
Spline
Resolution (nm)
Start Wavelength (nm)
End Wavelength (nm)
1 1.574 Extracted 0.06120 None None None 1.2 209.6 399.
2 2.195 Extracted 0.07729 None None None 1.2 209.6 399.
3 3.261 Extracted 0.24225 None None None 1.2 209.6 399.
4 3.871 Extracted 0.09939 None None None 1.2 209.6 399.
Legend
Spectra
Library Match
Spectrum Poi

Fig. S3

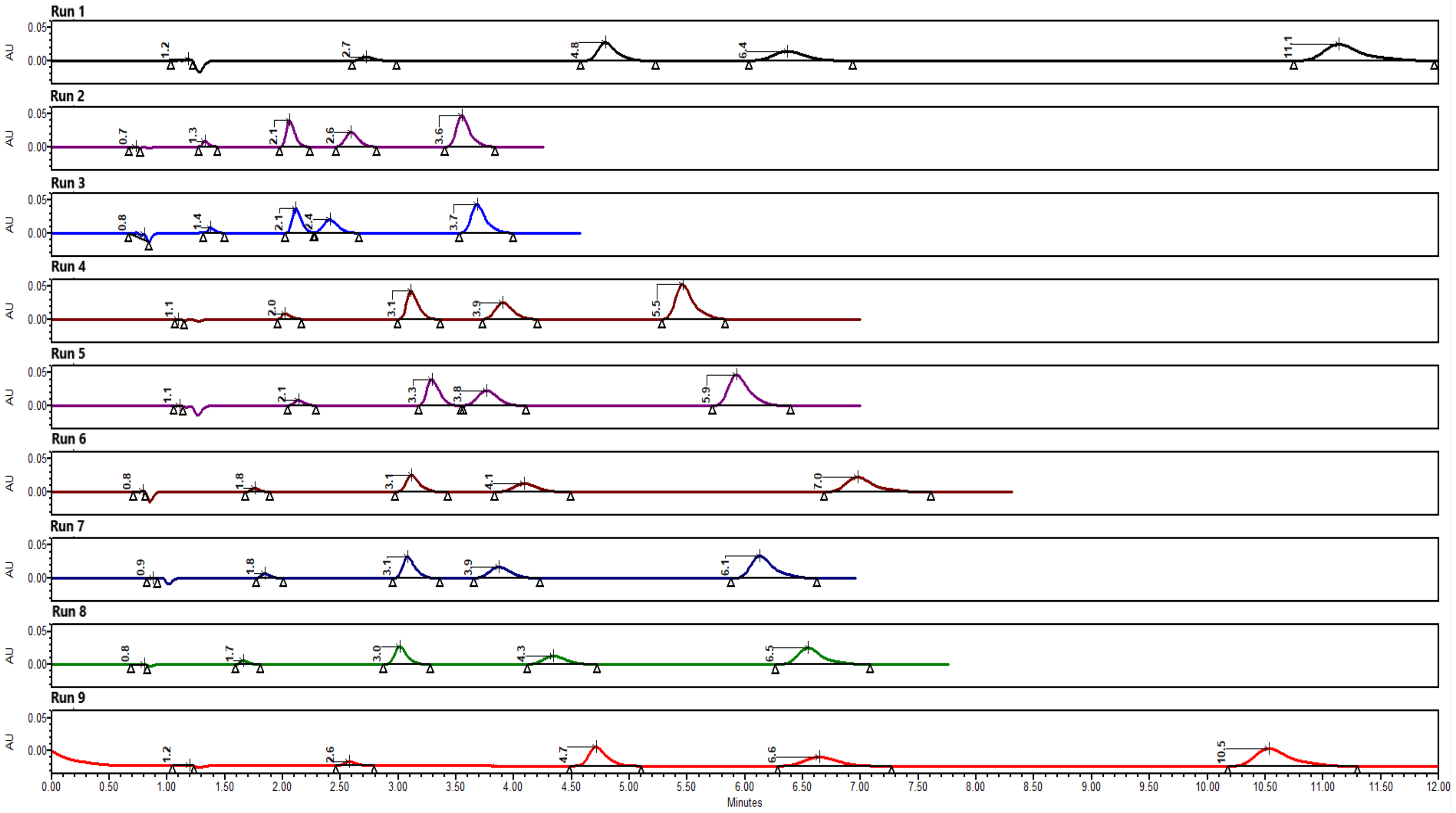

Run 1
Run 2
Run 3
Run 4
Run 5
Run 6
Run 7
Run 8
Run 9
AU
0.05
0.00
Minutes
0.00
0.50
1.00
1.50
2.00
2.50
3.00
3.50
4.00
4.50
5.00
5.50
6.00
6.50
7.00
7.50
8.00
8.50
9.00
9.50
10.00
10.50
11.00
11.50
12.00

Fig. S4

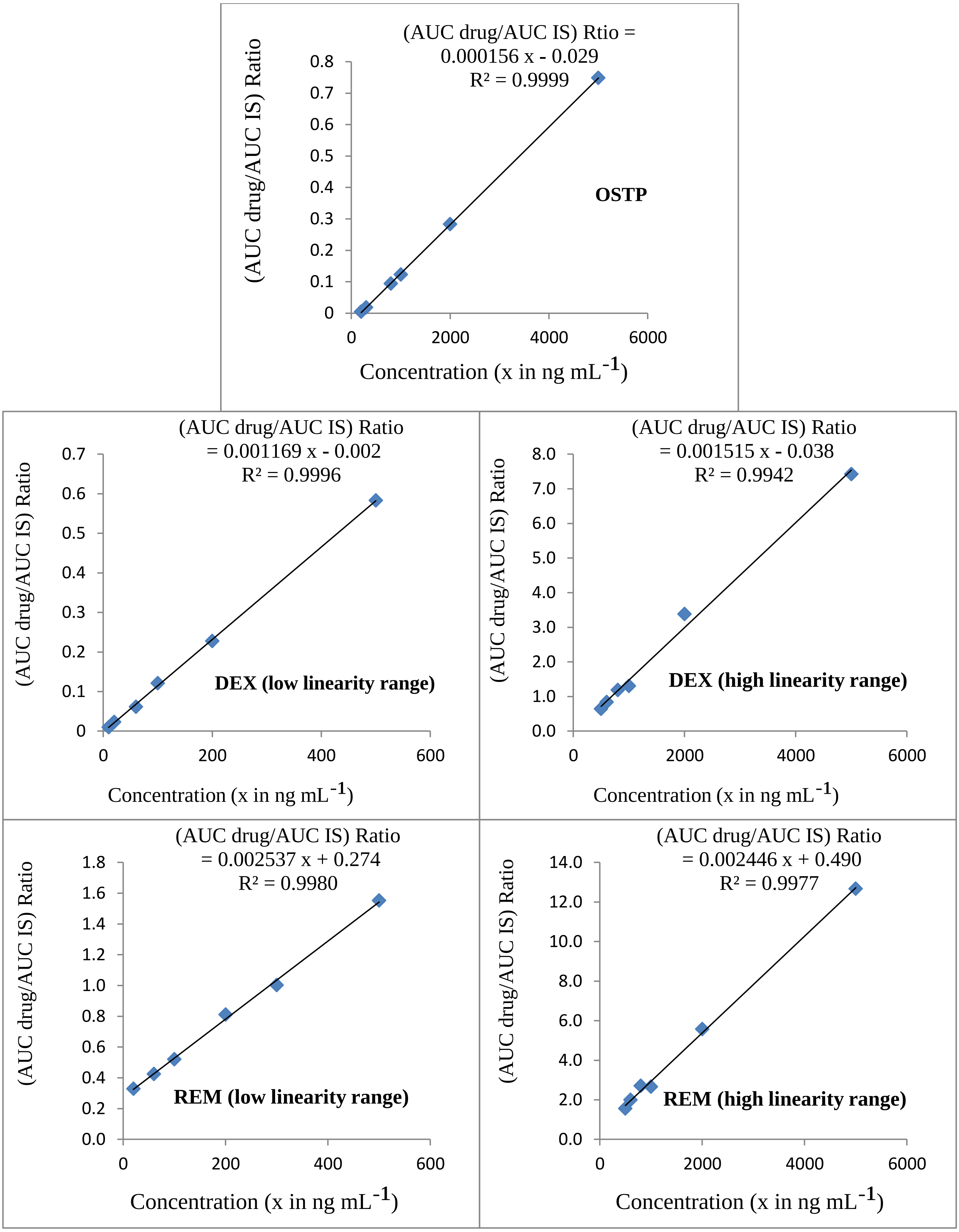
(AUC drug/AUC IS) Rtio =
0.000156 x - 0.029
R² = 0.9999
OSTP
(AUC drug/AUC IS) Ratio
Concentration (x in ng mL-1)
(AUC drug/AUC IS) Ratio
= 0.001169 x - 0.002
R² = 0.9996
DEX (low linearity range)
(AUC drug/AUC IS) Ratio
= 0.001515 x - 0.038
R² = 0.9942
DEX (high linearity range)
(AUC drug/AUC IS) Ratio
= 0.002537 x + 0.274
R² = 0.9980
REM (low linearity range)
(AUC drug/AUC IS) Ratio
= 0.002446 x + 0.490
R² = 0.9977
REM (high linearity range)

Fig. S5

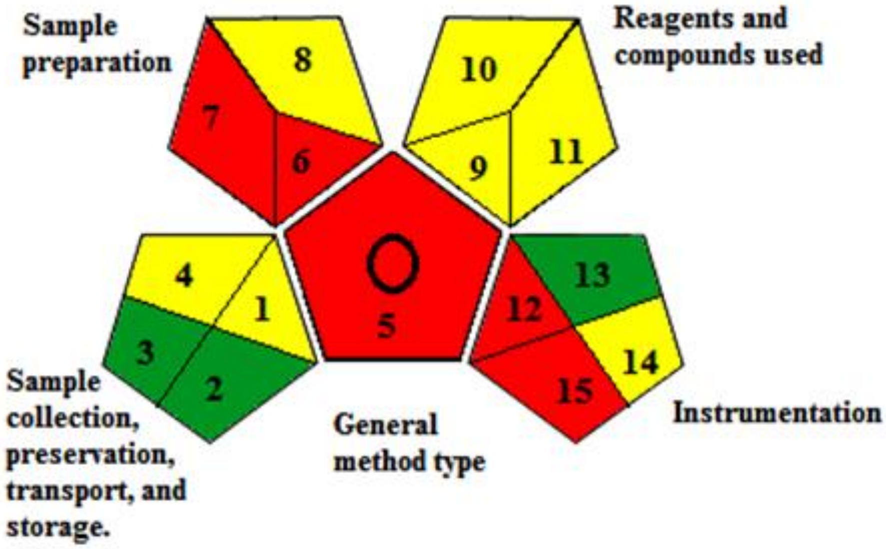

1- Collection.
2- Preservation.
3- Transport.
4- Storage.
5- Type of mrthod: direct or indirect.
6- Scale of extraction.
7- Solvents/ reagents used.
8- Additional treatment.
9- Reagent and solvents amount.
10- Health hazard.
11- Safety hazard.
12- Energy.
13- Occupational hazard.
14- Waste.
15- Waste treatment.
Circle in the middle of GAPI:Procedure for qualification and quantification

Fig. S6

Analytical Greenness report sheet

11/03/2023 00:42:07

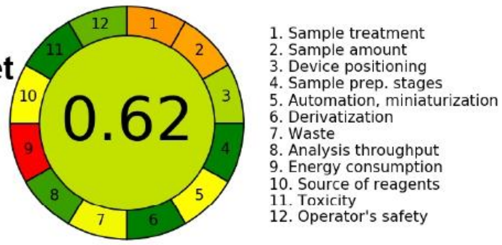

Criteria	Score	Weight
1. Direct analytical techniques should be applied to avoid sample treatment.	0.3	2
2. Minimal sample size and minimal number of samples are goals.	0.32	2
3. If possible, measurements should be performed in situ.	0.66	2
4. Integration of analytical processes and operations saves energy and reduces the use of reagents.	1.0	2
5. Automated and miniaturized methods should be selected.	0.5	2
6. Derivatization should be avoided.	1.0	2
7. Generation of a large volume of analytical waste should be avoided, and proper management of analytical waste should be provided.	0.53	2
8. Multi-analyte or multi-parameter methods are preferred versus methods using one analyte at a time.	0.89	2
9. The use of energy should be minimized.	0.0	2
10. Reagents obtained from renewable sources should be preferred.	0.5	2
11. Toxic reagents should be eliminated or replaced.	1.0	2
12. Operator's safety should be increased.	0.8	2

Table S1: Minitab response optimization and optimization plot of 2^3 full factorial design for RP-UPLC-PDA separation of oseltamivir phosphate/dexamethasone/daclatasvir dihydrochloride/remdesivir mixture.

Response optimization								
	**Goal**	**Lower**	**Target**	**Upper**	**Weight**	**Importance value**[b]	**Predicted Responses**	**Desirability (d)**[c]
$k'_{(OSTP)}$	Target	1.00	1.11	1.20	1	1	1.1196	0.80
$Rs_{2(DAC)}$	Target	3.10	3.21	3.30	1	1	3.2161	0.84
**Optimum Condition:**<br>**Methanol%** = 61.5%<br>**Flow rate (mL min^{-1})** = 0.25 mL min^{-1}<br>**Ammonium acetate (mM)** = 40 mM							**Composite Desirability (D)**[d] = 0.82	

Optimization plot[a]

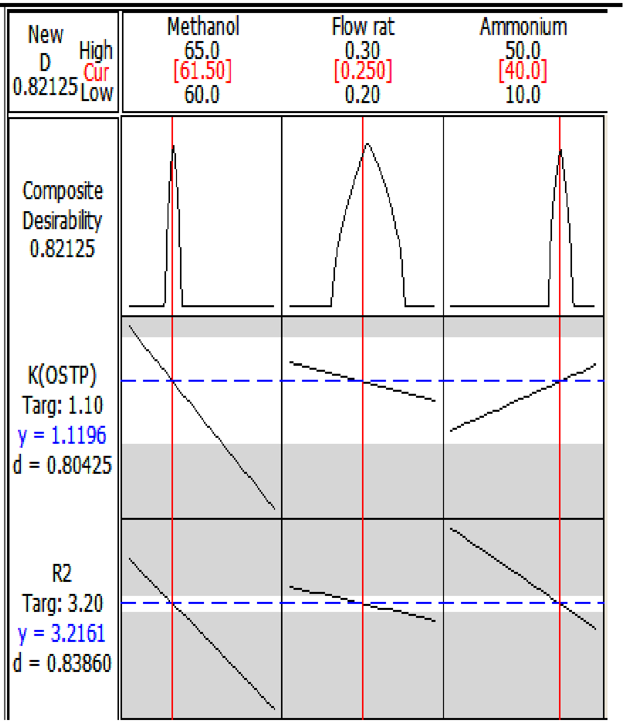

[a] The optimization plot showed the effect of each independent factor (columns) on the dependent responses (rows). The vertical red lines on the graph represent the optimum settings of the independent factors. The red numbers displayed at the top of the columns showed the optimum values of the independent factors. The horizontal dashed blue lines and numbers (y) represented the responses to the optimum settings.

[b] The importance value has a range of 0.1 to ten. Because all responses are equally significant, the default value of one was applied to each.

[c] Individual desirability (d) measures how well the conditions optimize a single response. Desirability is measured on a scale of zero to one.

[d] Composite desirability (D) measures how well the conditions optimize all responses in the set. Desirability is measured on a scale of zero to one.

Table S2*: Method development trials by RP-UPLC-PDA

Day #	Trial #	Method #	Drug (µg mL^{-1})	Injection volume (µL)	Methods' condition: Column temperature	Methanol%	Aqueous mobile phase%	Flow rate (mL min^{-1})	PDA (nm)	Diluting solvent	Results: Peaks Rt (min)	W (min)	T_{usp}	N_{usp}	*K*	R_S	AUC	Observation	Recommendation
1	1	1	OSTP (0.5)	10	25 ^{O}C	75.7% methanol	24.3% (8 mM AA (pH 7))	0.05	239	30% methanol	11.749	1.589	2.243	2808	1.611	-	4830402	Very long run time (40 min) and very broad peaks. Very tailed OSTP peak high resolution between DEX and REM peaks	Increase the flow rate to 0.1 mL min^{-1}. Try either: a- another IS b- Or decrease the retention time of DAC peak to be before REM peak.
			DEX (0.5)	10							15.770	2.01	1.287	4480	-	4.547	19907551		
			REM (0.5)	10							32.554	3.712	1.189	8460	-	14.333	39785768		
			DAC (IS) (0.5)	10							38.718	3.643	1.059	6300	-	3.661	8357513		
1	2	2	OSTP (0.5)	10	25 ^{O}C	75.7% methanol	24.3% (8 mM AA (pH 7))	0.10	239	30% methanol	4.148	0.368	1.776	10746	0.885	-	2212011	Run time decreased, the wide of the peaks decreased, *K*' decreased, and Rs decreased.	Increase the flow rate to 0.15 mL min^{-1}.
			DEX (0.5)	10							4.815	0.467	1.483	18210	-	4.229	10310948		
			REM (0.5)	10							7.046	0.766	1.433	19783	-	12.693	18320939		
1	3	3	OSTP (0.5)	10	25 ^{O}C	75.7% methanol	24.3% (8 mM AA (pH 7))	0.15	239	30% methanol	2.535	0.250	1.715	9729	0.690	-	1500275	Run time decreased and the wide of the peaks decreased.	Try DAC as IS again.
			DEX (0.5)	10							2.835	0.316	1.517	14825	-	2.938	7075675		
			REM (0.5)	10							3.797	0.411	1.466	15369	-	8.674	12026198		
1	4	3	OSTP (0.5)	10	25 ^{O}C	75.7% methanol	24.3% (8 mM AA (pH 7))	0.15	239	30% methanol	2.50	0.274	1.800	7057	0.667	-	1426618	The REM AUC increased (> trial.3). DAC (IS) overlapped with REM peak.	Decrease the flow rate to 0.1 mL min^{-1} to separate REM and DAC peaks.
			DEX (0.5)	10							2.803	0.317	1.564	12361	-	2.845	6332133		
			REM (0.5)	10							3.757	0.494	1.295	12760	-	8.296	13157139		
			DAC (IS) (0.5)	10															

1	5	2	OSTP (0.5)	10	25 ^{O}C	75.7% methanol	24.3% (8 mM AA (pH 7))	0.10	239	30% methanol	4.020	0.386	1.889	7390	0.827	-	2065534	The REM AUC increased (> trial.2). DAC (IS) still overlapped with REM peak.	Try favipiravir (FAV) as IS (it has fluorescence).
			DEX (0.5)	10							4.618	0.510	1.499	14067	-	3.329	9445837		
			REM (0.5)	10							6.709	0.676	1.539	14089	-	10.535	19800192		
			DAC (IS) (0.5)	10															
1	6	2	FAV (60)	10	25 ^{O}C	75.7% methanol	24.3% (8 mM AA (pH 7))	0.1	239	30% methanol	2.132	1.361	1.277	244	-0.94	-	26811387	Broad peak with very small *K* ’and N.	Try ledipasvir (LED) as IS (it has fluorescence) after blank (30% methanol).
1	7	2	Blank	10	25 ^{O}C	75.7% methanol	24.3% (8 mM AA (pH 7))	0.1	239	30% methanol	2.128	-	-	-	-	-	36247	-	Try LED as IS.
1	8	2	LED (10)	10	25 ^{O}C	75.7% methanol	24.3% (8 mM AA (pH 7))	0.1	239	30% methanol	2.097	-	-	-	-	-	53911	Led peak (bad in shape) eluted with the methanolic peak (after overlaying blank (trial 7) with trial 8).	Try FAV as IS again and try to increase its *K*’. Increase strength of AA to to mask silanol groups.
2	9	4	FAV (30)	0.1	25 ^{O}C	70% methanol	30% (30 mM AA (pH 6.05))	0.1	239	30% methanol	2.232	0.551	1.775	4518	0.015	-	75264	Very small *K*’.	Decrease methanol%.
2	10	5	FAV (30)	0.1	25 ^{O}C	65% methanol	35% (30 mM AA (pH 6.05))	0.1	239	30% methanol	2.249	0.447	1.671	4864	0.022	-	75731	Slight increase in *K*’.	Decrease methanol%.
2	11	6	FAV (30)	0.1	25 ^{O}C	55% methanol	45% (30 mM AA (pH 6.05))	0.1	239	30% methanol	2.288	0.463	1.629	5121	0.040	-	80513	Slight increase in *K*’.	Decrease methanol%. And to mask silanol groups and delay FAV elution, try either:

																			a- Increase AA strength<br>b- or decrease pH by phosphoric acid.<br>c- or both.
3	12	7	FAV (30)	0.1	25 ^{O}C	50% methanol	50% (50 mM AA (pH 6.90))	0.1	239	30% methanol	2.380	0.367	1.577	5082	0.082	-	92588	Slight increase in *K*'.	Decrease methanol%
3	13	8	FAV (30)	0.1	25 ^{O}C	40% methanol	60% (50 mM AA (pH 6.90))	0.1	239	30% methanol	2.475	0.432	1.637	5826	0.125	-	92513	Slight increase in *K*'. Effect of increasing AA strength is small on increasing of *K* 'even with decreasing pH.	Try 30 mM AA with low pH (pH 4) to enhance silanol groups masking effect and hence increasing *K*' De
3	14	9	FAV (30)	0.1	25 ^{O}C	40% methanol	60% (30 mM AA (pH 4.00))	0.1	239	30% methanol	3.112	0.382	1.449	10350	2.435	-	35349	*K* 'of FAV > 0.5 Unfortunately; this separation condition (MOH:AA=40:60, flow rate=0.1 mL min^{-1}) would delay OSTP, DEX, and REM very much.	Increase the flow rate to 0.2 mL min^{-1}
3	15	10	FAV (3)	10.0	25 ^{O}C	40% methanol	60% (30 mM AA (pH 4.00))	0.2	239	30% methanol	1.588	0.322	1.580	6114	0.397	-	43544	*K* 'of FAV decreased.	Decrease methanol % to 30% + Increase strength of

																			AA to 50 mM and adjust pH at 4 + Increase flow rate to 0.4 mL min^{-1}.
4	16	11	FAV (3)	0.1	25 ^{O}C	30% methanol	70% (50 mM AA (pH 4.00))	0.4	239	30% methanol	0.875	0.101	1.384	9034	0.471	-	7578	*K* ’of FAV increased.	Decrease methanol % to 20%
4	17	12	FAV (3)	0.1	25 ^{O}C	20% methanol	80% (50 mM AA (pH 4.00))	0.4	239	30% methanol	1.097	0.118	1.367	11057	0.813	-	8350	*K* ’of FAV increased.	Decrease methanol % to 5%
4	18	13	FAV (3)	0.1	25 ^{O}C	5% methanol	95% (50 mM AA (pH 4.00))	0.4	239	30% methanol	2.467	0.208	1.393	17292	2.766	-	8704	*K* ’of FAV increased to more than 2. Unfortunately; this separation condition (MOH:AA=5: 95, flow rate=0.4 mL min^{-1}) would delay OSTP, DEX, and REM	Try gradient method of separation but first try to reach the lowest methanol% that separate OSTP, DEX, and REM in 10 min.
4	19	14	OSTP (0.5)	10.0	25 ^{O}C	70% methanol	30% (50 mM AA (pH 4.00))	0.3	239	30% methanol	1.218	0.189	1.938	8688	0.664	-	8688	Low *K* ’and low run time (3 min)	Decrease methanol% to 65%.
			DEX (0.5)								1.675	0.211	1.398	11575	-	7.670	11575		
			REM (0.5)								2.485	0.25	1.308	11544	-	10.224	11544		
4	20	15	OSTP (0.5)	10.0	25 ^{O}C	65% methanol	35% (50 mM AA (pH 4.00))	0.3	239	30% methanol	1.443	0.143	1.490	11695	0.974	-	5305	Slight increase of *K* ’ and the run time increased to 4.5 min.	Decrease methanol% to 60%.
			DEX (0.5)								2.227	0.194	1.319	12161	-	11.394	32167		
			REM (0.5)								4.030	0.354	1.281	12287	-	15.590	52122		

4	21	16	OSTP (0.5)	10.0	25 ^{O}C	60% methanol	40% (50 mM AA (pH 4.00))	0.3	239	30% methanol	1.817	0.210	1.474	11235	1.486	-	6086	Increase of *K* 'and the run time increased to 7.5 min.	Increase the flow rate to 0.35 mL min^{-1}.
			DEX (0.5)								3.215	0.334	1.287	12733	-	14.960	32207		
			REM (0.5)								7.307	0.554	1.204	12822	-	21.590	50829		
4	22	17	OSTP (0.5)	10.0	25 ^{O}C	60% methanol	40% (50 mM AA (pH 4.00))	0.35	239	30% methanol	1.526	0.141	1.293	9944	1.388	-	4913	Slight decrease of *K* ' and the run time decreased to 6.5 min.	Increase the flow rate to 0.5 mL min^{-1}.
			DEX (0.5)								2.666	0.305	1.259	10842	-	13.623	27872		
			REM (0.5)								5.909	0.455	1.165	10905	-	19.472	42811		
4	23	18	OSTP (0.5)	10.0	25 ^{O}C	60% methanol	40% (50 mM AA (pH 4.00))	0.50	239	30% methanol	1.335	0.146	1.791	8463	1.363	-	4494	Slight decrease of *K* ' and the run time decreased to 5.5 min.	Decrease methanol% to 55% and the flow rate to 0.4 mL min^{-1}
			DEX (0.5)								2.311	0.334	1.231	9299	-	12.528	24219		
			REM (0.5)								5.049	0.447	1.195	9420	-	17.720	38315		
4	24	19	OSTP (0.5)	10.0	25 ^{O}C	55% methanol	45% (50 mM AA (pH 4.00))	0.40	239	30% methanol	1.761	0.264	1.373	9233	2.117	-	4517	Increase of *K* ' and the run time increased to 10.5 min.	Try gradient method to separate FAV (IS), OSTP, DEX, and REM mixture..
			DEX (0.5)								3.545	0.360	1.205	9914	-	16.189	24205		
			REM (0.5)								9.894	0.781	1.173	10041	-	23.288	37459		
4	25	20	FAV (0.6)	10.0	25 ^{O}C	*Time range: 0-2 min* (40% methanol:60% 50 mM AA (pH 4.00)) *Then, time range: 2-10 min* (60% methanol:40% 50 mM AA (pH 4.00))		0.40	239	30% methanol	0.781	0.147	1.528	5597	0.282	-	21725	Very low *K*', unstabilized baseline and high run time to 7.5 min.	Try another gradient method to low *K*.
			OSTP (0.5)								3.013	0.122	1.710	36728	-	41.200	5033		
			DEX (0.5)								4.178	0.226	1.281	36914	-	15.516	23720		
			REM (0.5)								6.939	0.343	1.230	22710	-	16.000	34363		
4	26	21	FAV (0.6)	10.0	25 ^{O}C	*Time range: 0-1.5 min* (40% methanol:60% 50		0.40	239	30% methan	0.779	0.132	2.270	10386	0.374	-	17216	Still very low *K*', very tailed	Try another gradient

			OSTP (0.5)			mM AA (pH 4.00)) *Then, time range: 1.5-1.55 min* (40% methanol:60% 50 mM AA (pH 4.00)) *Then, time range: 1.55-10 min* (60% methanol:40% 50 mM AA (pH 4.00))				ol	3.364	0.167	1.958	69347	-	61.67	4172	FAV peak, highly unstabilized baseline and high run time to 7.5 min..	method to low *K* and decrease tailing of FAV peak
			DEX (0.5)								4.461	0.248	1.524	38639	-	15.157	25515		
			REM (0.5)								7.145	0.344	1.252	24335	-	16.000	35606		
4	27	22	FAV (0.6)	10.0	25 ^{O}C	*Time rang: 0-2 min* (5% methanol:95% 50 mM AA (pH 4.00)) *Then, time range: 2-3 min* 30% methanol:70% 50 mM AA (pH 4.00)) *Then, time range:* 3-10 min (60% methanol:40% 50 mM AA (pH 4.00))		0.40	239	30% methanol	-	-	-	-	-	-	-	Highly unstabilized baseline with unidentified peaks. Gradient method to separate FAV (highly polar IS) from OSTP, DEX, and REM was difficult	Return to isocratic method. Use DAC as IS and elute it between DEX and REM peaks. Try methanol with water (pH 7) (60:40) as start to separate OSTP, DEX, DAC, and REM
			OSTP (0.5)								-	-	-	-	-	-	-		
			DEX (0.5)								-	-	-	-	-	-	-		
			REM (0.5)								-	-	-	-	-	-	-		
5	28	23	DEX (0.5)	10.0	25 ^{O}C	60% methanol	40% (Water (pH 7.00))	0.40	239	30% methanol	2.362	0.239	1.207	9362	2.937	-	24221	Good *K* '(>2) but run time would be less if DAC (IS) separated between DEX and REM. The OSTP peak was tailed and with relatively low N.	Try acidified water (pH 4.00) by phosphoric acid to increase OSTP and DAC ionization and masking of free silanol groups leading to decreasing the interaction of the ionized OSTP and DAC molecules
			OSTP (0.5)								4.530	0.298	1.894	8051	-	13.760	4235		
			REM (0.5)								5.398	0.487	1.200	9211	-	3.907	38239		
			DAC (0.5)								6.505	0.494	1.495	5426	-	3.991	6790		

																			with the free silanol groups This would led to: a- Decrease of OSTP tailing and elution of OSTP peak little earlier leading to decrease of the resolution between DEX and OSTP. b- Elution of DAC before REM and decreasing of the run time.
5	29	24	DEX (0.5)	10.0	25 ^{O}C	60% methanol	40% (Water (pH 4.00))	0.40	239	30% methanol	2.277	0.253	1.193	8789	2.795	-	24372	Good K '(> 2). DAC peak eluted before REM but its tailing was increased. OSTP peak still relatively tailed.	Try 10 mM AA (pH 4.00) by phosphoric acid to increase masking strength of silanol groups and decrease DAC and OSTP tailings.
			OSTP (0.5)								2.915	0.287	1.607	5296	-	4.863	4379		
			DAC (0.5)								3.817	0.387	2.155	3998	-	4.252	6757		
			REM (0.5)								5.088	0.48	1.189	8608	-	5.240	38096		
5	30	25	OSTP (0.5)	10.0	25 ^{O}C	60% methanol	40% (10 mM AA (pH 4.00))	0.40	239	30% methanol	1.359	0.134	1.394	7743	1.265	-	4283	OSTP eluted before DEX. OSTP and DAC tailings decreased.	Try 10 mM AA (pH 3.00) by phosphoric acid
			DEX (0.5)								2.465	0.254	1.201	8919	-	13.204	24047		
			DAC (0.5)								3.982	0.374	1.116	3658	-	8.432	7099		
			REM (0.5)								5.604	0.501	1.165	8985	-	6.570	38006		
5	31	26	DAC (0.5)	10.0	25 ^{O}C	60% methanol	40% (10 mM AA (pH	0.40	239	30% methanol	1.004	0.191	1.550	1111	0.673	-	8496	DAC eluted first with low *K*'	Try 10 mM AA (pH 5.00) by phosphoric
			OSTP								1.23	0.09	1.35	9558	-	2.44	4142		

			(0.5)				2.7.00))				0	9	8			3			acid
			DEX (0.5)								2.196	0.205	1.267	10428	-	13.915	23561		
			REM (0.5)								4.533	0.378	1.192	10364	-	17.383	36692		
5	32	27	OSTP (0.5)	10.0	25 ^{O}C	60% methanol	40% (10 mM AA (pH 4.7.00))	0.40	239	30% methanol	1.348	0.128	1.503	8163	1.247	-	4251	DAC eluted last one.	Try 50 mM AA (pH 5.00) by phosphoric acid.
			DEX (0.5)								2.267	0.259	1.297	9533	-	11.757	24344		
			REM (0.5)								4.978	0.464	1.265	9680	-	17.948	38257		
			DAC (0.5)								5.836	0.419	1.321	7059	-	3.466	6605		
5	33	28	OSTP (0.5)	10.0	25 ^{O}C	60% methanol	40% (50 mM AA (pH 5.00))	0.40	239	30% methanol	1.424	0.145	1.278	8127	1.373	-	4296	DAC separated after REM.	Try 50 mM AA (pH 7.00) by phosphoric acid.
			DEX (0.5)								2.338	0.295	1.148	9044	-	11.106	24459		
			REM (0.5)								5.184	0.387	1.075	9244	-	17.783	36932		
			DAC (0.5)								6.601	0.476	0.849	5452	-	5.418	6733		
5	34	29	OSTP (0.5)	10.0	25 ^{O}C	60% methanol	40% (50 mM AA (pH 7.00))	0.40	239	30% methanol	1.616	0.255	1.758	8447	1.693	-	4810	DAC separated after REM.	Try 50 mM AA (pH 4.00) by phosphoric acid.
			DEX (0.5)								2.291	0.316	1.272	9476	-	7.987	24490		
			REM (0.5)								4.962	0.484	1.273	9580	-	17.618	38103		
			DAC (0.5)								6.990	0.528	0.943	4139	-	6.679	5785		
5	35	19	OSTP (0.5)	10.0	25 ^{O}C	55% methanol	45% (50 mM AA (pH 4.00))	0.40	239	30% methanol	1.743	0.215	1.440	9058	2.113	-	4541	DAC separated before REM. AA (10-50 mM) at pH 4 was the most suitable range for separation.	Try flow rate 0.2 mL min^{-1}.
			DEX (0.5)								3.500	0.423	1.239	9692	-	16.051	24561		
			DAC (0.5)								5.100	0.577	1.308	2913	-	6.353	7437		
			REM (0.5)								9.783	0.766	1.172	10067	-	12.466	36989		

6	36	30	OSTP (0.5)	10.0	25 ^{O}C	55% methanol	45% (50 mM AA (pH 4.00))	0.20	239	30% methanol	3.805	0.230	1.156	15119	2.488	-	6835	Very long run time.	Try 65% methanol.
			DEX (0.5)								7.752	0.540	1.160	17438	-	21.443	51564		
			DAC (0.5)								13.345	0.939	1.103	7032	-	12.134	10795		
			REM (0.5)								23.753	1.452	1.080	16682	-	14.686	92570		
6	37	31	OSTP (0.5)	10.0	25 ^{O}C	65% methanol	30% (50 mM AA (pH 4.00))	0.20	239	30% methanol	2.126	0.309	1.464	2857	0.914	-	46469	Run time 7 min. Methanol % (60-65) was most suitable range.	Try flow rate 0.3 mL min^{-1}.
			DEX (0.5)								3.263	0.414	1.388	3667	-	5.990	310088		
			DAC (0.5)								3.766	0.620	1.208	2176	-	1.836	277931		
			REM (0.5)								5.916	0.755	1.473	4987	-	6.308	599395		
6	38	32	OSTP (0.5)	10.0	25 ^{O}C	65% methanol	30% (50 mM AA (pH 4.00))	0.30	239	30% methanol	1.382	0.182	1.480	2538	0.728	-	30224	Run time 4 min. *K* ’more than 0.7. Flow rate (0.2-0.3) mL min^{-1} was most suitable range.	Factorial design<br>FR= 0.2-0.3 mL min^{-1}<br>MOH% =60-65%<br>AA = 10-50-mM
			DEX (0.5)								2.117	0.248	1.346	3304	-	5.545	204497		
			DAC (0.5)								2.415	0.371	1.278	1674	-	1.519	173227		
			REM (0.5)								3.684	0.494	1.435	4014	-	5.269	393781		
7	39	33	OSTP (0.5)	10.0	25 ^{O}C	61.5% methanol	38.5% (40 mM AA (pH 4.00))	0.25	239	60% methanol	1.817	0.222	1.495	5998	1.163	-	34378	The final mixture was in 60% methanol instead of 30% methanol.<br>Good system suitability parameters	Try at column temperatures (30 ^{o}C).
			DEX (0.5)								2.994	0.360	1.244	8138	-	10.102	254667		
			DAC (0.5)								3.900	0.582	1.204	2877	-	4.189	220571		
			REM (0.5)								5.831	0.616	1.154	9932	-	7.217	473971		

																		were observed.	
7	40	34	OSTP (0.5)	10	30 ^{O}C	61.5% methanol	38.5% (40 mM AA (pH 4.00))	0.25	239	60% methanol	1.715	0.187	1.478	4474	1.091	-	34569	Enhancements of system suitability parameters were observed.	Try at column temperatures (45 ^{o}C).
			DEX (0.5)								2.818	0.289	1.194	8525	-	9.652	250923		
			DAC (0.5)								3.704	0.486	1.057	3098	-	4.484	218976		
			REM (0.5)								5.570	0.451	1.124	10090	-	7.530	467932		
7	41	35	OSTP (0.5)	10	45 ^{O}C	61.5% methanol	38.5% (40 mM AA (pH 4.00))	0.25	239	60% methanol	1.598	0.190	1.429	6273	0.837	-	36907	Enhancements of system suitability parameters were observed.	Try at column temperatures (50 ^{o}C).
			DEX (0.5)								2.270	0.258	1.300	8341	-	7.246	251055		
			DAC (0.5)								3.394	0.319	1.181	9050	-	9.091	218619		
			REM (0.5)								4.065	0.339	1.125	10993	-	4.425	463209		
7	42	36	OSTP (0.5)	10	50 ^{O}C	61.5% methanol	38.5% (40 mM AA (pH 4.00))	0.25	239	60% methanol	1.574	0.140	1.394	8722	0.789	-	38968	Enhancements of system suitability parameters were observed.	Apply in human plasma in fluoride, EDTA, and fluoride-EDTA.
			DEX (0.5)								2.194	0.200	1.368	10775	-	7.925	252806		
			DAC (0.5)								3.260	0.239	1.246	11442	-	10.071	218391		
			REM (0.5)								3.870	0.303	1.241	12787	-	4.603	473558		

*Notes on Table S2: It was suggested to choose favipiravir (FAV)[1], DAC[2], or ledipasivir (LED)[3] as the internal standard (IS). Different conditions were used to develop the method. The studied drugs (OSTP, DEX, and REM) with DAC as IS were tried to be simultaneously separated by a mobile phase consisting of a high amount of methanol percentage (MOH%) with 8 mM ammonium acetate at pH 7. The flow rate was set at 0.05 mL min^{-1}, and the column oven temperature was 25 ^{o}C (Table S2, trial 1). It was observed that the order of eluted peaks was OSTP, DEX, REM, and then DAC. The run time was very long, and all resulted peaks were very broad. Also, OSTP was very tailed (USP peak tailing, T = 2.2). So, it was recommended to increase the flow rate to reduce the run time (Table S2, trials 2–5). It was observed that the run time and the width of the peaks were reduced. But unfortunately, REM and DAC (IS) peaks overlapped. So, FAV and LED were tried as IS (Table S2, trials 6–18). LED peak was very bad

in shape and was overlapped with the solvent peak, while FAV showed promising results. FAV showed good system suitability parameters at separation conditions of MOH with 50 mM ammonium acetate (pH 4) at a ratio of 5:95, flow rate of 0.4 mL min^{-1}, and column oven temperature of 25 ^oC (Table S2, trial 18). The purpose of the high strength (50 Mm) with low pH (4) of ammonium acetate was to mask the free silanol groups and hence delay FAV elution (capacity factor, $K' = 2.8$) as its log P is very small (nearly 0.5) (Table S3), and to reduce T of FAV as ammonium acetate can improve peak shape not only by deactivating the free silanol groups but also by forming ion pairs with the analyte[4]. Unfortunately, this separation condition of 5% MOH with 95% ammonium acetate would dramatically delay the elution of OSTP, DEX, and REM as they have high log P (more than 1) (Table S3). So, the idea of developing a gradient method to elute the highly polar FAV (IS) with the less polar compounds (OSTP, DEX, and REM) in a reasonable time (less than 10 min) was recommended. This gradient method would start with a low MOH percentage in the mobile phase to elute FAV (IS) and end with a high MOH percentage to elute the studied drugs. Different trials were used to develop this gradient method (Table S2, trials 19–27), but a highly unstabilized baseline with a very low FAV capacity factor ($K' \approx 0.3$) and badly shaped peaks were identified.

So, returning to the isocratic method was recommended. DAC was tried again as IS. It has a good log P (Table S3) to be eluted with the studied drugs under isocratic conditions. But the main problem was its overlapping with REM (Table S2, trials 2–5). So, different trials were used to solve this problem (Table S2, trials 28–35). Deionized water (pH 7) as an aqueous mobile phase with MOH at a ratio of 60:40 was first tried as a mobile phase. At a flow rate of 0.4 mL min^{-1} and a column oven temperature of 25 ^oC, the order of the eluted peaks was DEX, OSTP, REM, and then DAC (Table S2, trial 28). DEX had a good capacity factor ($K' = 2.9$), but the OSTP peak was tailed (T = 1.9) and had a high USP resolution (R = 13.8). DAC (IS) was eluted after the tested drugs, increasing the run time to 7 min. So, it was recommended to try the acidified, deionized water (pH 4) with phosphoric acid (Table S2, trial 29) to increase OSTP and DAC ionization (Table S3) and at the same time mask the free silanol groups in the stationary phase. This would lead to decreasing the interaction of the ionized OSTP and DAC molecules with free silanol groups. And hence, OSTP peak tailing and resolution would be decreased as it would be eluted a little earlier. Also, ionized DAC molecules would be eluted before the unionized REM molecules, leading to a shorter run time. At Trial 29 (Table S2), it was observed that OSTP peak tailing was relatively decreased, but DAC peak tailing was increased. So, it was suggested to use 10 mM ammonium acetate at pH 4 (Table S2, trial 30) to increase the masking effect of the free silanol groups and hence decrease the peak tailings. At Trial 30 (Table S2), it was observed that DAC and OSTP tailings were decreased and that OSTP was eluted before DEX due to lower interactions between the ionized OSTP and free silanol groups. So the final order at Trial 30 (Table S2) was OSTP, DEX, DAC (IS), and then REM with a separation condition of MOH: ammonium acetate (pH 4) =40:60, flow rate 0.4 mL min^{-1}, and column oven temperature of 25 ^oC. Different strengths of ammonium acetate with different pHs (3-7) were tried (Table S2, trials 31-35). It was observed that the retention times of OSTP and DAC were greatly affected by pH of the ammonium acetate aqueous mobile phase. So, it was concluded that ammonium acetate (10-50 mM) as an aqueous mobile phase solution adjusted at pH 4 with phosphoric acid showed the most suitable peak order (OSTP, DEX, DAC, and then REM) with good system suitability parameters. Finally, Trials 35–38 (Table S2) were tested in order to identify the factorial design factors and their levels.

Table S3: Physical characters of oseltamivir phosphate, dexamesasone, remdesivir, favipiravir, daclatasvir dihydrochloride, and ledipasivir.

Drug		Water solubility	The log P (log Kow)	Acidic function group			Basic function group		
				**Pka (strongest acidic)**	**Status at pH [4.0 − 4.5]**	**Status at pH [7.0 − 7.5]**	**Pka (strongest basic)**	**Status at pH [4.0 − 4.5]**	**Status at pH [7.0 − 7.5]**
**Studied drugs**	**Oseltamivir phosphate**	0.686 mg mL^{-1}	1.30	14.03	Unionized	Unionized	9.31	Ionized	Partially ionized (ionized molecules > unionized molecules)
	**Dexamesasone**	0.0505 mg mL^{-1}	1.93	12.42	Unionized	Unionized	-3.3	Unionized	Unionized
	**Remdesivir**	0.339 mg mL^{-1}	2.10<br>3.20	10.23	Unionized	Unionized	0.65	Unionized	Unionized
**Suggested IS**	**Favipiravir**	8.7 mg mL^{-1}	0.49	9.39	Unionized	Unionized	-3.7	Unionized	Unionized
	**Daclatasvir dihydrochloride**	0.00283 mg mL^{-1}	3.47	3.82	Partially ionized (ionized molecules > unionized molecules)	ionized	6.09	Ionized	Partially unionized (unionized molecules > ionized molecules)
	**Ledipasivir**	0.00388 mg mL^{-1}	5.98<br>7.18	11.33	Unionized	Unionized	5.29	Ionized	Unionized

Table S4: 2^3 FFD experimental factorial designs with centerpoint experiment and their dependent responses at 239 nm for RP-UPLC-PDA separation of the oseltamivir phosphate/dexamethasone/daclatasvir dihydrochloride/remdesivir mixture.

Design order				Experimental factorial design[a]			Dependent responses															
							OSTP				DEX				DAC				REM			
Std Order	Run Order	CenterPt	Blocks	(A) MOH%[b]	(B) AA conc.(mM)[c]	(C) FR (mL min^{-1})[d]	Rt_1[e]	k'[f]	T_1[g]	N_1[h]	Rt_2	R_{S1}	T_2	N_2	Rt_3	R_{S2}	T_3	N_3	Rt_4	R_{S3}	T_4	N_4
5	1	1	1	60.0	0.20	50	2.725	1.29928	1.30515	2866.24	4.794	8.07261	1.43938	4314.64	6.369	3.78127	1.21980	2434.15	11.140	8.44638	1.54815	5971.21
4	2	1	1	65.0	0.30	10	1.330	0.81439	1.54043	2926.33	2.061	5.98997	1.40748	3639.16	2.594	2.91881	1.23988	2273.73	3.551	4.35265	1.41591	4629.54
8	3	1	1	65.0	0.30	50	1.379	0.86538	1.47254	2581.70	2.116	5.58419	1.34415	3358.00	2.409	1.49790	1.27928	1653.88	3.684	5.32188	1.42866	4111.62
2	4	1	1	65.0	0.20	10	2.022	0.84179	1.55656	3253.74	3.112	6.35626	1.46698	4355.29	3.907	3.21165	1.25666	2904.21	5.461	5.27701	1.49880	6159.23
6	5	1	1	65.0	0.20	50	2.137	0.92355	1.40005	2970.91	3.292	6.12612	1.38461	3798.96	3.766	1.74387	1.20283	2175.26	5.926	6.39896	1.46736	5178.11
7	6	1	1	60.0	0.30	50	1.759	1.22131	1.28069	2508.42	3.118	7.48805	1.41765	3488.13	4.091	3.17800	1.21182	1812.51	6.976	6.92075	1.49126	4286.95
9	7	0	1	62.5	0.25	30	1.846	1.09538	1.46309	2582.64	3.082	6.88088	1.43267	3701.38	3.874	2.79187	1.22124	1969.12	6.130	6.24761	1.51934	4852.27
3	8	1	1	60.0	0.30	10	1.661	1.06818	1.45993	2618.97	3.015	8.05513	1.39301	3834.83	4.344	4.52675	1.23445	2141.11	6.549	5.64896	1.44799	4713.61
1	9	1	1	60.0	0.20	10	2.576	1.15534	1.45473	2850.76	4.713	8.65063	1.40850	4352.15	6.645	4.55598	1.20951	2423.15	10.532	6.89374	1.54522	5858.00

[a] Three replicates of each experimental design were performed.
[b] Methanol% (v/v) (low level 73% and high level 77%).
[c] Ammonium acetate concentration. (mM) (low level 5 mM and high level 20 mM).
[d] Flow rate (mL min^{-1}) (low level 0.03 and high level 0.07).
[e] Peak retention time.
[f] Peak capacity factor.
[g] Peak tailing was evaluated at 5% (USP).
[h] A drug peak's theoretical plate count.

Table S5: Regression analyses for $k'_{(OSTP)}$ and $R_{S2(DAC)}$ versus methanol% (v/v), flow rate (mL min^{-1}), and ammonium acetate (mM) using 2^3 FFD with centerpoint

Predictor (Independent variable)	$k'_{(OSTP)}$			$R_{S2(DAC)}$		
	**Coefficient**	**P-value**[a]	**Data subsetting lack of fit test (Curvature)**	**Coefficient**	**P-value**[a]	**Data subsetting lack of fit test (Curvature)**
**Constant (Intercept)**	5.16710	0.000[b]	-	25.64900	0.000[b]	-
**Methanol% (v/v)**	-0.06495	0.000	No evidence	-0.33349	0.000	No evidence
**Flow rate (mL min^{-1})**	-0.62680	0.091	No evidence	-2.92800	0.146	No evidence
**Ammonium acetate (mM)**	0.00269	0.016	No evidence	-0.03133	0.001	No evidence
**R^2**[c]	96.4%			96.8%		
**Adjusted R^2**[d]	94.3%			95.0%		
**Predicted R^2**[e]	90.50%			91.28%		

[a] The P-value determines the appropriateness of rejecting the null hypothesis in a hypothesis test. P-values range from 0 to 1. A commonly used α-level value is 0.05. If the p-value of a test statistic is less than α-level, the null hypothesis is rejected.

[b] In practice, the p-value for the intercept term is unimportant. However, the intercept term in the model would still be kept even if the p-value isn't less than a-level (0.05).

[c] R^2 (R-squared) is the percentage of response variable variation that is explained by its relationship with one or more predictor variables. In general, the higher the R^2, the better the model fits your data. R^2 is always between 0 and 100%. It is also known as the coefficient of determination or multiple determinations (in multiple regressions).

[d] Adjusted R^2 is the percentage of response variable variation that is explained by its relationship with one or more predictor variables, adjusted for the number of predictors in the model. This adjustment is important because the R^2 for any model will always increase when a new term is added. A model with more terms may appear to have a better fit simply because it has more terms. However, some increases in R^2 may be due to chance alone. The adjusted R^2 is a useful tool for comparing the explanatory power of models with different numbers of predictors. The adjusted R^2 will increase only if the new term improves the model more than would be expected by chance. It will decrease when a predictor improves the model less than expected by chance.

[e] Predicted R^2 is used in regression analysis to indicate how well the model predicts responses for new observations, whereas R^2 indicates how well the model fits your data. Predicted R^2 can prevent overfitting the model and can be more useful than adjusted R^2 for comparing models because it is calculated using observations not included in model estimation. Overfitting refers to models that appear to explain the relationship between the predictor and response variables for the data set used for model calculation but fail to provide valid predictions for new observations. Predicted R^2 is calculated by systematically removing each observation from the data set, estimating the regression equation, and determining how well the model predicts the removed observation. The predicted R^2 ranges between 0 and 100%. Larger values of predicted R^2 suggest models of greater predictive ability.

Table S6: The preliminary stability study of the tested drugs in in different human plasma kits (K_3EDTA, fluoride, and fluoride-EDTA)

Concentration in plasma (ng mL^{-1})	Plasma type	OSTP 1st hour (Mean ± SD[a], RSD %)	OSTP 2nd hour (Mean ± SD[a], RSD %)	DEX 1st hour (Mean ± SD[a], RSD %)	DEX 2nd hour (Mean ± SD[a], RSD %)	REM 1st hour (Mean ± SD[a], RSD %)	REM 2nd hour (Mean ± SD[a], RSD %)
**300**	**Fluoride**	96.78 ± 4.70, 4.86	81.51 ± 2.00, 2.46	104.22 ± 2.40, 2.30	77.85 ± 1.86, 2.39	111.18 ± 1.50, 1.35	90.47 ± 3.74, 4.13
	**EDTA**	93.49 ± 2.77, 2.96	81.21 ± 2.43, 2.99	100.41 ± 1.28, 1.27	91.71 ± 2.04, 2.23	96.31 ± 3.31, 3.44	85.81 ± 2.26, 2.64
	**Fluoride + EDTA**	**108.18 ± 2.28, 2.11**	**101.24 ± 2.55, 2.52**	**102.90 ± 1.87, 1.82**	**98.53 ± 3.10, 3.14**	**105.35 ± 3.22, 3.05**	**98.52 ± 1.97, 2.00**
**1000**	**Fluoride**	95.33 ± 1.88, 1.97	85.50 ± 2.93, 3.43	93.87 ± 3.10, 3.30	71.72 ± 2.47, 3.44	111.72 ± 2.05, 1.83	98.28 ± 2.55, 2.59
	**EDTA**	92.64 ± 3.02, 3.25	81.08 ± 1.74, 2.15	97.78 ± 2.48, 2.54	88.77 ± 3.07, 3.45	93.08 ± 2.60, 2.79	88.07 ±2.62, 2.97
	**Fluoride + EDTA**	**102.28 ± 1.98, 1.93**	**93.46 ± 3.10, 3.31**	**107.95 ± 3.06, 2.84**	**101.38 ± 3.37, 3.33**	**110.93 ± 3.00, 2.70**	**94.92 ± 1.02, 1.07**

[a] Each result is the mean of 3 results in percentage ± their standard deviation in percentage.

Table S7: Application of the proposed method for the analysis of oseltamivir phosphate/dexamethasone/remdesivir mixture in human plasma (n = 6).

**Oseltamivir phosphate**			
**200-5000 (ng mL^{-1})**			
**Nominal Conc.[a], ng mL^{-1}**	**Conc. found[b] ± SD, ng mL^{-1}**	**Accuracy (Mean, %)[b, c]**	**Precision (CV, %)**
200	217.33 ± 0.64	108.66	0.30
300	303.59 ± 22.29	101.20	7.34
800	791.09 ± 3.14	98.89	0.40
1000	978.37 ± 9.98	97.84	1.02
2000	2007.97 ± 33.13	100.40	1.65
5000	5001.66 ± 17.89	100.03	0.36

**Dexamethasone**							
**10-500 (ng mL^{-1})**				**500-5000 (ng mL^{-1})**			
**Nominal Conc., ng mL^{-1}**	**Conc. found[b] ± SD, ng mL^{-1}**	**Accuracy (Mean, %)[b, c]**	**Precision (CV, %)**	**Nominal Conc., ng mL^{-1}**	**Conc. found[b] ± SD, ng mL^{-1}**	**Accuracy (Mean, %)[b, c]**	**Precision (CV, %)**
10	10.56 ± 0.72	105.61	6.86	500	446.72 ± 29.29	89.35	6.56
20	21.77 ± 1.66	108.88	7.61	600	579.12 ± 2.28	96.52	0.39
60	54.73 ± 4.71	91.21	8.61	800	806.98 ± 4.72	100.87	0.59
100	105.49 ± 6.42	105.49	6.08	1000	883.77 ± 10.38	88.38	1.17
200	196.65 ± 5.08	98.33	2.58	2000	2255.74 ± 42.53	112.79	1.89
500	500.79 ± 9.78	100.16	1.95	5000	4927.67 ± 33.31	98.55	0.68

**Remdesivir**							
**20-500 (ng mL^{-1})**				**500-5000 (ng mL^{-1})**			
**Nominal Conc., ng mL^{-1}**	**Conc. found[b] ± SD, ng mL^{-1}**	**Accuracy (Mean, %)[b, c]**	**Precision (CV, %)**	**Nominal Conc., ng mL^{-1}**	**Conc. found[b] ± SD, ng mL^{-1}**	**Accuracy (Mean, %)[b, c]**	**Precision (CV, %)**
20	21.50 ± 1.71	107.48	7.95	500	434.51 ± 21.04	86.90	4.84
60	59.15 ± 7.32	98.58	12.38	600	616.86 ± 30.26	102.81	4.91
100	97.01 ± 4.25	97.02	4.38	800	905.78 ± 24.56	113.22	2.71
200	211.53 ±13.38	105.77	6.33	1000	886.89 ± 17.27	88.69	1.95
300	286.96 ± 15.04	95.65	5.24	2000	2076.22 ± 15.05	103.81	0.73
500	503.85 ± 20.29	100.77	4.03	5000	4979.74 ± 20.53	99.60	0.41

[a] Conc.: Concentration.
[b] The mean of six determinations.
[c] The accuracy should be within ± 20% of the nominal concentration at the LLOQ and within ±15% at all the other levels.

Table S8: Accuracy and precision results for the determination of oseltamivir phosphate/dexamethasone/remdesivir mixtures in human plasma by the proposed method.

Drug name		Oseltamivir phosphate				Dexamethasone								Remdesivir							
Linearity range		200-5000 (ng mL^{-1})				10-500 (ng mL^{-1})				500-5000 (ng mL^{-1})				20-500 (ng mL^{-1})				500-5000 (ng mL^{-1})			
Conc. taken in mixture[a]		200 (LLOQ)	300 (LQC)	1000 (MQC)	3000 (HQC)	10 (LLOQ)	40 (LQC)	80 (MQC)	300 (HQC)	500 (LLOQ)	600 (LQC)	1000 (MQC)	3000 (HQC)	20 (LLOQ)	40 (LQC)	80 (MQC)	300 (HQC)	500 (LLOQ)	600 (LQC)	1000 (MQC)	3000 (HQC)
Within run[b]	Found, %	108.46	92.62	98.98	98.15	113.50	85.45	102.22	112.23	95.20	96.60	89.52	105.41	95.65	101.32	89.32	108.83	82.08	97.26	87.36	109.43
		95.00	105.66	97.41	93.19	99.25	102.63	108.64	110.56	83.48	96.85	88.12	96.74	101.55	103.88	89.68	100.83	88.77	104.05	90.64	101.82
		108.50	105.31	97.12	91.21	104.09	106.49	110.50	110.53	89.36	96.10	87.49	92.75	99.91	117.23	102.54	102.42	89.86	107.12	88.07	96.52
		99.00	99.36	105.48	91.57	90.80	102.30	93.79	101.61	92.18	96.50	108.37	97.31	95.65	108.50	97.38	108.94	92.20	115.00	111.32	107.14
		109.03	90.49	96.45	98.06	89.50	94.83	105.53	103.38	100.38	111.10	102.07	89.54	101.55	97.70	94.83	93.98	102.20	118.82	90.90	84.88
	**Accuracy[c] (Mean, %)**	**104.00**	**98.69**	**99.09**	**94.44**	**99.43**	**98.34**	**104.13**	**107.66**	**92.12**	**99.43**	**95.11**	**96.35**	**105.73**	**94.75**	**103.00**	**97.37**	**91.02**	**108.45**	**93.66**	**99.96**
	**SD, %**	**6.55**	**7.02**	**3.69**	**3.43**	**9.91**	**8.35**	**6.58**	**4.81**	**6.32**	**6.53**	**9.52**	**5.97**	**7.54**	**5.54**	**6.24**	**4.36**	**7.29**	**8.61**	**9.99**	**9.79**
	**Precision[d] (CV, %)**	**6.30**	**7.11**	**3.72**	**3.63**	**9.97**	**8.49**	**6.32**	**4.47**	**6.86**	**6.56**	**10.01**	**6.19**	**7.13**	**5.85**	**6.06**	**4.48**	**8.01**	**7.94**	**10.67**	**9.80**
Between run[e]	Found, %	108.66	101.20	97.84	94.18	105.61	98.19	107.12	111.11	89.34	96.52	88.38	98.30	107.48	93.85	104.03	95.65	86.90%	102.81	88.69	102.59
		107.22	99.85	101.92	89.41	110.10	89.03	115.76	102.73	91.18	112.00	110.33	88.70	86.15	99.69	106.68	101.55	89.77%	114.36	102.81	85.36
		97.83	86.78	98.93	99.89	102.00	99.79	100.12	99.99	94.00	96.17	102.76	99.85	98.08	96.42	102.11	99.91	105.82%	114.70	89.41	100.59
	**Accuracy[c] (Mean, %)**	**104.57**	**95.94**	**99.56**	**94.49**	**105.90**	**95.67**	**107.67**	**104.61**	**91.51**	**101.56**	**100.49**	**95.61**	**97.23**	**96.65**	**104.27**	**99.04**	**94.16**	**110.62**	**93.64**	**96.18**
	**SD, %**	**5.88**	**7.96**	**2.11**	**5.25**	**4.06**	**5.80**	**7.83**	**5.79**	**2.34**	**9.04**	**11.15**	**6.04**	**10.69**	**2.93**	**2.29**	**3.05**	**10.19**	**6.77**	**7.95**	**9.43**
	**Precision[d] (CV, %)**	**5.63**	**8.30**	**2.12**	**5.56**	**3.83**	**6.07**	**7.27**	**5.54**	**2.56**	**8.90**	**11.10**	**6.32**	**11.00**	**3.03**	**2.20**	**3.08**	**10.83**	**6.12**	**8.49**	**9.80**

[a] Each mixture contains equal concentrations of OSTP, DEX, and REM.
[b] Each result is the average of five determinations.
[c] The acceptance criteria were ± 15 % of the nominal concentration's values for QC samples and with ± 20 % for LLOQ.
[d] The acceptance criteria should not exceed 15 % for QC samples except for LLOQ which should be less than 20 %.
[e] Each result is the average of nine determinations over three days (n=3 per day).

Table S9: The matrix effect (%ME), extraction efficiency (%RE), and process efficiency (%PE) and their IS normalized values for the tested drugs in fluoride-EDTA human plasma

Concentration in plasma (ng mL^{-1})	Drug	Matrix effect (%ME) (Mean ± SD[a] %, RSD %)	IS normalized matrix effect % (Mean ± SD[a] %, RSD %)	Extraction efficiency (%RE) (Mean ± SD[a] %, RSD %)	IS normalized extraction efficiency % (Mean ± SD[a] %, RSD %)	Process efficiency (%PE) (Mean ± SD[a] %, RSD %)	IS normalized Process efficiency % (Mean ± SD[a] %, RSD %)
**300**	**OSTP**	89.72 ± 1.18, 1.31	88.87 ± 2.11, 2.37	93.06 ± 4.07, 4.37	97.19 ± 5.56, 5.72	83.50 ± 4.21, 5.04	86.29 ± 2.84, 3.29
	**DEX**	94.27 ± 1.54, 1.63	93.43 ± 4.69, 5.02	88.53 ± 1.81, 2.05	92.42 ± 1.90, 2.05	83.47 ± 2.85, 3.41	86.34 ± 4.38, 5.08
	**REM**	96.96 ± 2.41, 2.49	96.08 ± 4.49, 4.67	94.40 ± 3.23, 3.43	98.54 ± 2.72, 2.76	91.52 ± 3.28, 3.58	94.67 ± 4.75, 5.02
**1000**	**OSTP**	89.40 ± 1.62, 1.82	91.22 ± 0.96, 1.05	94.64 ± 0.85, 0.90	96.94 ± 1.21, 1.25	84.60 ± 0.89, 1.05	88.43 ± 1.32, 1.49
	**DEX**	85.95 ± 1.79, 2.08	87.70 ± 0.55, 0.63	94.68 ± 1.57, 1.66	96.98 ± 1.55, 1.60	81.39 ± 2.52, 3.10	85.05 ± 0.96, 1.12
	**REM**	96.88 ± 2.36, 2.44	98.86 ± 2.02, 2.04	98.45 ± 1.43, 1.45	100.85 ± 1.27, 1.26	95.41 ± 3.66, 3.84	99.71 ± 3.17, 3.18

[a] Each result is the mean of four results prepared from blank plasma from four different sources (plasma A, B, C, and D) in percentage ± their standard deviation in percentage.

Table S10: The penalty points of the proposed method according to the analytical Eco-Scale per sample

Requirements per sample			Simultaneous separation of OSTP, DEX, and DAC in human plasma	Simultaneous separation of OSTP and DEX in human plasma	Simultaneous separation of DEX and REM in human plasma	Determination of OSTP in human plasma	Determination of DEX in human plasma	Determination of REM in human plasma
**Reagents**	Methanol	≈ 27 mL	12	12	12	12	12	12
	Ammonium acetate	0.0013 gm	1	1	1	1	1	1
	Diethyl ether	2 mL	4	4	4	4	4	4
	Human plasma	0.5 mL	0	0	0	0	0	0
	OSTP	0.010 gm	1	1	–	1	–	–
	DEX	0.010 gm	2	2	2	–	2	–
	DAC (IS)	0.010 gm	0	0	0	0	0	0
	REM	0.010 gm	1	–	1	–	–	1
**Instruments**	UPLC[a].	Energy ≤ 0.1 kWh	0	0	0	0	0	0
	Vortex mixer	Energy ≤ 1.5 kWh	1	1	1	1	1	1
	Centrifuge	Energy ≤ 1.5 kWh	1	1	1	1	1	1
	Water path	Energy ≤ 1.5 kWh	1	1	1	1	1	1
	Ultra low freezer	Energy > 1.5 kWh	2	2	2	2	2	2
	Occupational hazard	Analytical process hermetization	0	0	0	0	0	0
	Waste	Less than 4 mL, no treatment	6	6	6	6	6	6
**Total penalty points**			32	31	31	29	30	29
**Analytical eco-scale total score [b]**			68	69	69	71	70	71

[a] Ultralow pressure liquid chromatography.
[b] If the score is more than 75, it represents excellent green analysis.
If the score is more than 50, it represents acceptable green analysis.
If the score is less than 50, it represents inadequate green analysis.

Equations (supplementary materials)

$k' = (t_R - t_m)/t_m$ Equation (S1)

Where;

k' is the analyte peak's capacity factor, commonly known as the mass distribution ratio (D_m) or retention factor (k, R). Its suggested value is [0.5–10.0][5], where t_R is the analyte peak retention time (min) and t_m is the solvent front peak retention time (min).

$R_S = 2\left(\frac{\mathrm{Peak}_{(n+1)} t_R - \mathrm{Peak}_{(n)} t_R}{W_{(n+1)} + W_{(n)}}\right)$ Equation (S2)

Where;

The recommended value for R_S is greater than 1.5, which represents the resolution of two consecutive peaks (the higher the R_S, the greater the separation). Peak t_R represents peak retention time, n represents peak serial number, and W represents peak width.

$T = W_{5\%}/2f$ Equation (S3)

Where;

T is the analyte peak's tailing factor, commonly known as the symmetry factor (*As*). Its recommended range is [0.8–2][6]. $W_{5\%}$is the width of the analyte peak at 5% height, and f is the distance from the peak maximum to the leading edge of the peak measured at 5% of the peak height from the baseline.

$N = 5.54\left(\frac{t_R}{W50\%}\right)^2$ Equation (S4)

Where;

The number of theoretical plates of the analyte peak, N or NTP, is recommended to be [1000–20,000] plates (the greater the N, the better the efficiency);t_R stands for retention time (min); and $W_{50\%}$ stands for analyte peak width at 50% height.

[1] Drugbank, Favipiravir, DrugBank Online, 2023.
[2] Drugbank, Daclatasvir dihydrochloride, DrugBank, 2023.
[3] Drugbank, Ledipasvir, DrugBank, 2023.
[4] A. Cazenave Gassiot, B. Boughtflower, J. Caldwell, L. Hitzel, C. Holyoak, S. Lane, P. Oakley, F. Pullen, S. Richardson, G. Langley, Effect of increasing concentration of ammonium acetate as an additive in supercritical fluid chromatography using CO2-methanol mobile phase, J. Chromatogr. A, 1216 (2009) 6441-6450.
[5] M. Wadie, S.M. Mostafa, S.M. El.Adl, M.S. Elgawish, Development and validation of a new, simple-hplc method for simultaneous determination of sofosbuvir, daclatasvir and ribavirin in tablet dosage form, IOSR J. Pharm. & Biol. Sci., 12 (2017) 60-68.
[6] U.S. FDA, Reviewer guidance, validation of chromatographic methods, Center for drug evaluation and research, 1994.
